# Noninvasive Nonlinear Optical Computational Histology

**DOI:** 10.1002/advs.202308630

**Published:** 2023-12-14

**Authors:** Binglin Shen, Zhenglin Li, Ying Pan, Yuan Guo, Zongyi Yin, Rui Hu, Junle Qu, Liwei Liu

**Affiliations:** ^1^ Key Laboratory of Optoelectronic Devices and Systems of Guangdong Province and Ministry of Education College of Physics and Optoelectronic Engineering Shenzhen University Shenzhen 518060 China; ^2^ China–Japan Union Hospital of Jilin University Changchun 130033 China; ^3^ Shaanxi Provincial Cancer Hospital Xi'an 710065 China; ^4^ Shenzhen University General Hospital Shenzhen 518055 China

**Keywords:** cancer diagnosis, deep learning, multiphoton microscopy, nonlinear optical imaging, Stimulated Raman scattering microscopy

## Abstract

Cancer remains a global health challenge, demanding early detection and accurate diagnosis for improved patient outcomes. An intelligent paradigm is introduced that elevates label‐free nonlinear optical imaging with contrastive patch‐wise learning, yielding stain‐free nonlinear optical computational histology (NOCH). NOCH enables swift, precise diagnostic analysis of fresh tissues, reducing patient anxiety and healthcare costs. Nonlinear modalities are evaluated, including stimulated Raman scattering and multiphoton imaging, for their ability to enhance tumor microenvironment sensitivity, pathological analysis, and cancer examination. Quantitative analysis confirmed that NOCH images accurately reproduce nuclear morphometric features across different cancer stages. Key diagnostic features, such as nuclear morphology, size, and nuclear‐cytoplasmic contrast, are well preserved. NOCH models also demonstrate promising generalization when applied to other pathological tissues. The study unites label‐free nonlinear optical imaging with histopathology using contrastive learning to establish stain‐free computational histology. NOCH provides a rapid, non‐invasive, and precise approach to surgical pathology, holding immense potential for revolutionizing cancer diagnosis and surgical interventions.

## Graphical abstract

This paper presents stain‐free nonlinear optical computational histology (NOCH) for rapid, non‐invasive fresh tissue analysis. By fusing rich intrinsic signals of nonlinear optical imaging with tailored contrastive deep learning, NOCH digitally stains label‐free images into realistic virtual histological slides. Quantitative validations across different cancer types and stages confirm accurate reproduction of essential pathological features, providing histopathology without cumbersome staining workflows.

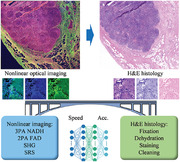

## 1 Introduction

Cancer represents a major global health burden with over 19 million new cases and 10 million deaths worldwide in 2020.^[^ ^1^, ^2^ ^]^ Early detection and diagnosis are critical for improving patient outcomes. Research consistently shows that over 80% of cancer patients undergo surgery after diagnosis.^[^ ^3^, ^4^, ^5^ ^]^ This underscores the importance of rapid and accurate pathological analysis of fresh tissue to guide surgical interventions and enhance the chances of a cure. However, traditional frozen section pathological examinations require the expertise of experienced pathologists and typically take 30 to 60 min to provide diagnostic results from tissue samples.^[^ ^6^, ^7^, ^8^, ^9^ ^]^ This time frame falls short of the real‐time demands of clinical practice, leading to prolonged waiting times for patients, inefficient use of medical resources, and increased socioeconomic costs.^[^ ^10^ ^]^ Therefore, there is an urgent need for the development of a fast, accurate, and responsive pathological diagnostic technology. Such technology would enable precise tumor resection during surgery, reducing the risk of postoperative recurrence and ultimately improving the overall cure rate for tumors.

As one of the promising stain‐free histopathological techniques,^[^ ^6^, ^7^, ^8^, ^9^ ^]^ label‐free nonlinear optical imaging (NLOI) holds the potential to swiftly offer rich bio‐molecular information for in‐depth and comprehensive analyses of the heterogeneous tumor microenvironment.^[^ ^11^, ^12^, ^13^ ^]^ While previous studies demonstrate the potential for label‐free live imaging using individual modalities, each mode provides only partial intrinsic tissue information that could be enhanced by incorporating complementary data from additional modes to enable more comprehensive and informative imaging.^[^ ^13^ ^]^ Recent studies further showed the power of integrating contrast modalities for visualizing important cancer‐associated processes including tumor cell migration, angiogenesis, and microvesicle enrichment without exogenous stains.^[^ ^6^, ^9^, ^14^ ^]^ However, the application of this technology in further preclinical and clinical studies faces two major limitations. First, the selection and combination of numerous nonlinear signals that provide valuable insights into the tumor microenvironment require further investigation. These signals originate from different endogenous biological macromolecules, offering high complementarity and contrast. However, there is a lack of an appropriate and rational approach for determining that modal choices are most advantageous for specific applications. Second, NLOI data can be challenging for pathologists and surgeons who prefer examining pathological tissues and assessing cancerization based on familiar imaging morphology akin to hematoxylin‐eosin (H&E) staining. The lack of histopathological interpretation, owing to the low cytoplasm‐nucleus contrast, and complex quantitative approaches^[^ ^13^, ^14^, ^15^ ^]^ constrain the application of label‐free NLOI in biomedical research and clinical pathology.

The concept of virtual histology^[^ ^5^, ^16^, ^17^, ^18^, ^19^, ^20^, ^21^ ^]^ holds the potential to address these challenges by merging the non‐invasiveness of NLOI with the interpretability of H&E staining, effectively mitigating the limitations of both techniques. This method, primarily based on convolutional neural networks (CNNs) and generative adversarial networks (GANs), have paved the way for rapid histological examination of various tissue types, significantly accelerating the diagnostic process.^[^ ^22^ ^]^ In this study, we establish a computational bridge between label‐free NLOI and histopathology using contrastive patch‐wise learning. By implementing self‐contrastive learning within image patches or cross‐contrastive learning between patches, we present noninvasive nonlinear optical computational histology (NOCH), which harnesses the power of biophotonic NLOI signals obtained from unstained tissues. We exemplify NOCH through stimulated Raman scattering (SRS) images for neurosurgical pathology of brain tumors and multiphoton (MP) images for clinicopathological analysis of ovarian cancer. These translations enhanced tumor microenvironment sensitivity, enabled pathological analysis, and improved cancer detection. We conduct comprehensive evaluations of different nonlinear modalities to ascertain their distinct contributions to histopathological translation and cancer diagnosis. Our quantifications of nuclear distributions and staining components demonstrate good preservation of key diagnostic features such as nuclear morphology, size, distance, and nuclear‐cytoplasmic contrast. Moreover, we tested the trained NOCH model on other pathological tissues, providing initial evidence of the generalization capability of the contrastive patch‐wise learning network. Our approach leverages NOCH to improve the interpretability of NLOI signals, provide richer cancer‐related information, accelerate the speed of pathological diagnosis without compromising diagnostic accuracy.

## 2 Results

### 2.1 NOCH via Contrastive Patch‐Wise Learning

**Figure 1:**
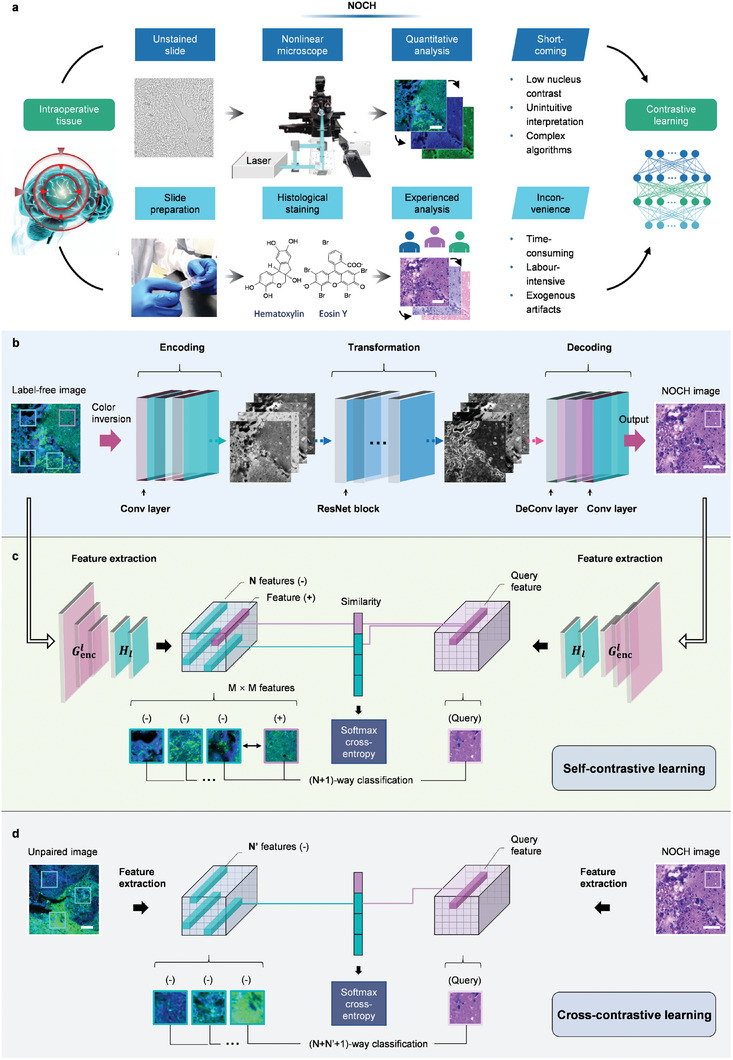
NLOI, NOCH and histopathological staining workflows and the network principle. a) The label‐free multi‐contrast NLOI imaging procedure for quantitative analysis, offering insights into the tumor microenvironment. In contrast, the standard histopathological procedure is employed for experienced examination and structural annotation. The NLOI and histopathological images were trained in the contrastive patch‐wise learning network. b) The basic architecture of the network. The generator was an encoder‐decoder structure, complemented by cascaded ResNet blocks. c) Mutual information maximized by the multilayer, patch‐wise self‐contrastive learning. This approach involves feature extraction and (N+1)‐way classification, facilitating the comparison of features between the input and temporary output data. Self‐contrastive similarity is established among positive (+) and negative (‐) patches within the same image, as well as with the query patch. d) Cross‐contrastive learning utilizing external negatives from other images through a momentum encoder. $G_{enc}^{l}$, the *l*‐th layer of the generative encoder; ***H***_***l***_: two‐layer multilayer perceptron (MLP) network. Scale bar, 100 µm.

Label‐free NLOI signals offer significant potential for providing rich structural and functional insights despite the lack of histopathological interpretation and the intricacies of associated analysis algorithms. Conventional histopathological examination, involving various steps such as tissue fixation, dehydration, and staining, is a time‐consuming and externally invasive procedure. NOCH can connect NLOI and traditional staining techniques to mitigate these challenges (**Figure** **1**a). This AI approach allows for the direct use of unstained tissues in pathological examination. However, a big challenge stems from the fact that unstained and stained tissues are typically closely sectioned and may not perfectly correspond. This discrepancy can lead to inaccurate pixel loss calculations when utilizing a straightforward translation discriminator. To tackle this issue effectively, we have implemented a contrastive unregistered patch‐wise learning translation network to minimize disparities, as illustrated in Figure 1b and detailed in Experimental Section.

The contrastive learning focuses on the commonalities between the input and output domains, e.g. the structures and shapes of the organ, while maintaining invariant differences, e.g. the textures. The entire input and output images, as well as their corresponding patches should share content. For instance, when presented with a patch displaying the normal cortex of the brain in the output virtually stained image, the aim is to establish a stronger association with the corresponding cortex in the input nonlinear image, as opposed to other features like tumors (as depicted in Figure 1c). The content consistency, rather than appearance, was realized by maximizing the mutual information of patches extracted from the input NLOI and output NOCH images.^[^ ^23^ ^]^ The core principle of self‐contrastive learning is to link two randomly sampled patches: the query and its positive counterpart, as opposed to other N featured patches, which were referred to as negatives, within the same image. The likelihood of selecting the positive example over the negatives is determined by solving a classification problem involving N+1 categories. The network employs noise contrastive estimation (NCE) to train an encoder, bringing related signals closer together and computing the self‐contrastive loss (Experimental Section). This approach ensures the consistency of features and overcomes the limitations of the cycle‐consistent methods.

Alternatively, cross‐contrastive learning takes external negatives from other images using a momentum encoder (Figure 1d). These external negatives may exhibit more significant differences from the current positive feature. Both negatives originating from the same image as the positive and those from other images are utilized to solve the classification problem and calculate the contrastive loss. To assess the effectiveness of both contrastive learning methods, we conducted a performance comparison on brain tissues by translating the SRS images into histological images.

### 2.2 SRS‐Based NOCH for Brain Tumor

**Figure 2:**
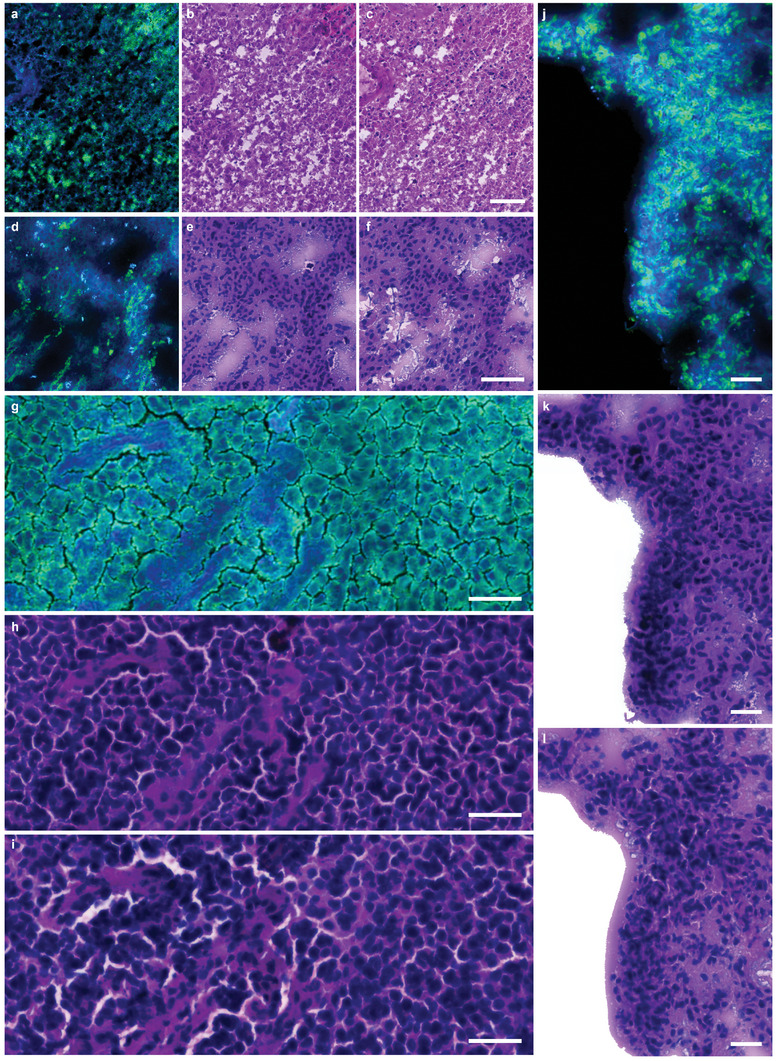
Label‐Free neurosurgical pathology using NOCH. a–c,g–i) Glioblastoma IV; d–f,j–l) Oligoastrocytoma II–III. The unstained SRS images a,d,g,j) were merged by protein (green) and lipid (blue) image. The NOCH network inferences b,e,h,k) closely mirror the corresponding pathological morphology observed in the H&E histopathology. c,f,i,l). Scale bar, 100 µm in (a–f) 40 µm in (g–l).

The brain, a crucial organ, poses significant challenges when it comes to the precise removal of tumors without causing damage to vital brain structures. Traditional histopathology offers valuable insights into tumor areas but presents limitations such as the inability to be performed repeatedly during surgery and the time‐consuming nature of the process. SRS microscopy enables rapid, non‐invasive mapping of lipids and proteins in fresh brain specimens. Through the application of contrastive patch‐wise learning, we transformed these molecular maps into images that closely approximate traditional pathology. Our analysis encompassed the large‐scale specimens with a diverse range of brain tumors,^[^ ^24^ ^]^ achieving NOCH transformation using the SRS image data. By meticulously evaluating the label‐free SRS data and comparing it to the clinical gold standard of H&E histopathology, we found that many essential diagnostic markers for glioblastoma and oligoastrocytoma, including nuclear density and glioma morphology, were effectively preserved (**Figure** **2**). These nuclear features cannot be obtained simply by linear color remapping. The virtual histological images underwent review by an experienced neuropathologist and neurosurgeon, who identified comparable and interpretable histological features akin to those observed in H&E histological slides.

We conducted a comparative analysis of the performance of self‐contrastive learning, cross‐contrastive learning, and the widely used Cycle‐GAN^[^ ^25^ ^]^ (Figure S1, Supporting Information). We trained the Cycle‐GAN network to facilitate image transformation between the two domains while maintaining style consistency through cycle‐consistent loss (as explained in Note S1, Supporting Information). However, the results obtained from Cycle‐GAN exhibited noticeable inconsistencies in histological styles, leading to evident histological translation artifacts (HTA) in the oligoastrocytoma and focal cortical dysplasia tissues as observed in Figure S1b (Supporting Information). Pseudo holes were generated despite training the network in a paired data mode (with even more divergent results observed in the unpaired mode). These limitations may be attributed to the strict bi‐directional convertibility enforced by cycle consistency, which might not always align with the characteristics of SRS and H&E images. In contrast, the outcomes of self‐contrastive learning (Figure S1c, Supporting Information) and cross‐contrastive learning (Figure S1d, Supporting Information) exhibited significantly fewer HTA and displayed a higher degree of similarity in histopathological morphology concerning the adjacent H&E slices (Figure S1e, Supporting Information). For a large‐field brain image (Figure S2, Supporting Information), contrastive learning can appropriately stain SRS, producing NOCH image with clear component margins, while the pathological representation generated by Cycle‐GAN diverged from that of H&E staining. This aids in the identification and selective resection of pathological tissue while preserving adjacent normal regions during surgery.

### 2.3 Contribution of SRS Modalities to NOCH

We assessed the impact of using a single SRS modality versus multiple modalities on virtual staining accuracy using contrastive learning. Comparisons in Figure S3 (Supporting Information) show that while virtual staining by a single SRS channel ≈2845 or 2930 cm^−1^ is feasible, ensuring consistent staining accuracy across different tissue slices in a single training epoch is challenging. In contrast, NOCH based on bimodal SRS images exhibits remarkably reliable staining accuracy across diverse tissue types (Figure 2; Figures S2 and S3, Supporting Information). This represents a significant improvement over single modal NOCH. Key histological attributes including nuclear counts and sizes remained well‐matched between bimodal NOCH outputs and real H&E staining (Figure S3h, Supporting Information). Moreover, the enhanced nuclear contrast in the bimodal NOCH images analogous to non‐adjacent histology sections (Figure S4, Supporting Information) is useful for analyzing nuclear morphology. Nuclear shape analysis provides insights into architectural changes associated with pathology. Thus, expanding the inputs to include both protein and lipid SRS channels provides complementary biochemical information about tissue architecture, enabling robust virtual H&E translation. Our results underscore the advantages of leveraging multiparametric SRS data rather than single channels alone for generating accurate and consistent computational histopathology. Thereby, NOCH based on SRS imaging and contrastive learning exhibits potential in assessing nuclear histomorphology and generating valuable marker‐free references for neuropathology, offering the ability to evaluate tissue heterogeneity in human brain tumors.

### 2.4 MP‐Based NOCH for Ovarian Cancer

**Figure 3:**
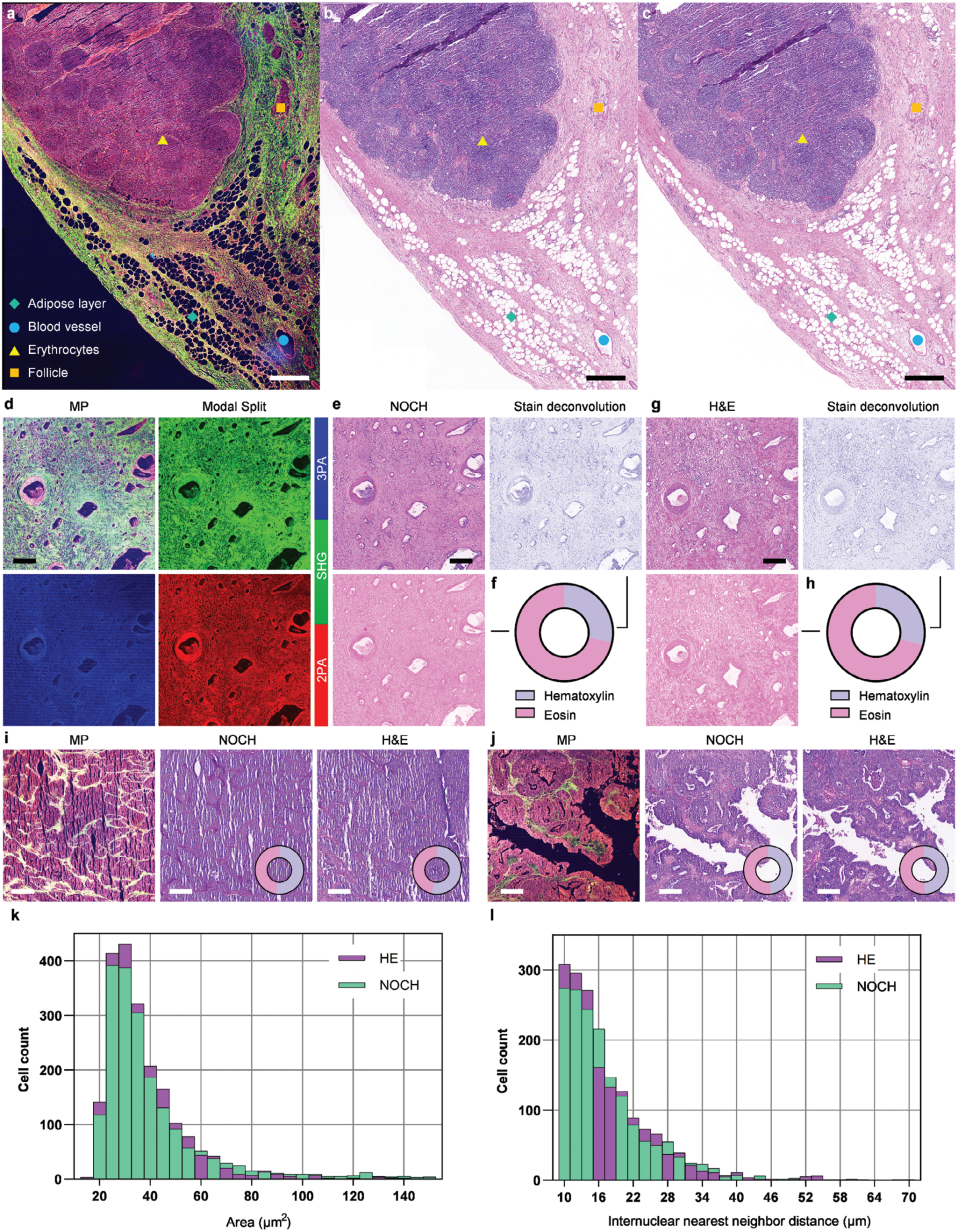
Label‐free ovarian pathology using NOCH. a) Large‐field MP image merged by 3PA NADH (blue), SHG (green), and 2PA FAD (red) captured using a MP microscope at 1,140 nm excitation. b) Inferred image using self‐contrastive learning. c) Histopathology of the adjacent slice of the normal ovarian tissue. d) MP image of Stage IIIC of ovarian cancer, with monochromatic image for each nonlinear modality. e) NOCH image and stain deconvolution for hematoxylin and eosin components, with the donut graph (f) showing the component ratio. g) H&E image and stain deconvolution for hematoxylin and eosin components, with the donut graph (h) showing the component ratio. i) Left to right: MP, NOCH, and H&E images of Stage IIC. j) Left to right: MP, NOCH, and H&E images of Stage IA. k) Histogram distribution of nuclear cross‐sectional areas in NOCH and H&E images of Stage IIIC. l) Histogram distribution of internuclear nearest neighbor distances in NOCH and H&E images of Stage IIIC. Scale bar, 500 µm in (a–c) and 200 µm in (d–j).

Different from SRS, which relies on Raman scattering of vibrational modes of CH bonds, MP imaging relies on non‐resonant processes that provide contrast for biological molecules and structures of flavin adenine dinucleotide (FAD) and nicotinamide adenine dinucleotide (NADH). We acquired a large dataset of multimodal MP modalities, including three‐photon autofluorescence (3PA) of NADH, SHG of collagen fibers, and two‐photon autofluorescence (2PA) of FAD from human ovarian specimens for NOCH using the contrastive learning approach. After the network inference, the characteristic components of the normal tissue, such as atretic follicles, blood vessels, erythrocytes, and the adipose layer in the MP images (**Figure** **3**a) were effectively transformed to a pathological morphology (Figure 3b), which exhibits strong agreement with real H&E morphology (Figure 3c). This verifies the consistency of the tissue structural transformation in the virtual staining.

Subsequently, we extended our approach to encompass various cancer stages (Figure 3d) of clinicopathologic ovarian tissues. We employed our network to translate these data into histopathological images (Figure 3d–j). The consistency observed between the virtually stained (Figure 3e) and histologically stained images (Figure 3g) can be attributed to the effective patch‐wise self‐contrastive learning applied to the unregistered unstained and stained images. Furthermore, the hematoxylin and eosin components, derived from the NOCH images, closely matched that of the H&E reference. Both deconvolutions exhibit a remarkably similar hematoxylin proportion, with 28% for NOCH and 29% for H&E. This consistency holds true even in different cancer stages, such as Stage IIC (Figure 3i) and IA (Figure 3j). Thus, contrastive learning is able to produce distinct staining patterns for various cancer stages, and the morphology and decomposition of these stains closely resemble real histological results.

Furthermore, we quantified the distribution of nuclear sizes (Figure 3k) and inter‐nuclear distances (Figure 3l) in the histological images of the ovarian cancer tissues. In both virtual and authentic histological images, nuclei were identified and extracted through a nuclei detection method tailored for H&E images, incorporating star‐convex shape priors.^[^ ^26^ ^]^ Subsequently, we derived the nearest neighbor distances by utilizing the X and Y coordinates of the nuclei centroids. Notably, the nuclear sizes with confidence interval (CI) = 39.62, 41.65 for NOCH and 36.55, 37.97 for H&E and inter‐nuclear distances with CI = 15.52, 16.20 for NOCH and 15.24, 15.93 for H&E exhibited high congruence between both sets of images. Slight variations in cell counts and nuclear cross‐sectional areas were expected, as we examined adjacent sections taken at different axial positions, rather than the exact same section. In contrast, the cycle‐consistent method exhibited significant deviations in nuclear segmentation in some tissues compared to contrastive learning and H&E (Figure S5a–d, Supporting Information), displaying notably smaller nuclear size and spacing distributions than their corresponding values (Figure S5e,f, Supporting Information). These results highlight the efficacy of our approach in achieving accurate computational histology for ovarian tissues. Expert validation of the virtual histological images was conducted by two experienced gynecological oncologists. Their assessment confirmed the existence of equivalent and interpretable histological features in the H&E histological slides, indicating the potential utility of these virtual images for clinical diagnosis.

The pretrained NOCH model for ovarian histology translation was applied to breast and liver tissues without retraining to demonstrate transfer learning capabilities (Note S2, Supporting Information). Despite misalignments from examining adjacent sections, virtual H&E slides of breast biopsy samples closely matched ground truth, faithfully rendering features identified via SHG and 2PA FAD (Figure S6a–c, Supporting Information). Similarly, MP images of liver tissue were rapidly converted to accurate histopathological morphology, with cancer‐associated features closely resembling real H&E staining (Figure S6d–g, Supporting Information). These results firmly establish the ability of the network to generalize and computationally stain diverse tissue types after training on one dataset.

### 2.5 Contribution of MP Modalities to NOCH

To assess the impact of different MP modalities on virtual staining and diagnosis, we conducted a comprehensive analysis using enumeration, permutation, and combination of the three nonlinear modalities (Figure S7, Supporting Information). We used contrastive learning to generate virtually histopathological results using various combinations of MP modalities, each yielding distinct levels of histological authenticity and HTA. For instance, significant histological disparities, when compared to H&E histology, were evident in the NOCH images generated from modalities with limited information content. These modalities included single 3PA, single SHG, and the 3PA‐SHG combination, primarily due to their inability to provide adequate tissue structural information.

**Figure 4:**
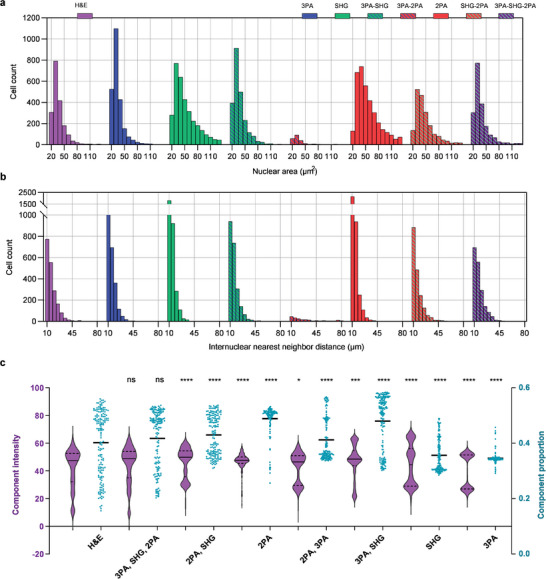
Quantification of modality combinations on NOCH and diagnosis. a) Histogram distribution of nuclear cross‐sectional areas (a) and internuclear nearest neighbor distances (b) in NOCH images of different modalities and H&E images of Stage IIIC. c) Component intensity of hematoxylin and eosin (violin plot) and hematoxylin proportion (scatter plot) derived from the H&E and NOCH images. *n* = 160. Asterisks denote significance levels tested with Kolmogorov‐Smirnov test; ns: no significantly difference for *p* > 0.05, ^*^ for *p* < 0.05 and ^****^ for *p* < 0.0001.

To quantify the contributions of the seven MP modality combinations, we computed nuclear size and spacing distributions for cancer Stage IIIC (Figure 3d–g) using different metrics (**Figure** **4**). The produced NOCH images by single modality (3PA, SHG, or 2PA) exhibited considerable deviations in nuclear size distribution (Figure 4a) and inter‐nuclear spacing distribution from H&E staining (Figure 4b). The introduction of dual modalities notably improved performance. However, the combination of the three modalities yielded the most consistent nuclear characteristics, closely approaching the that of the H&E histology (Figure 4a,b). Thereby, NOCH virtually stained tissue in a non‐invasive, label‐free manner without perturbing native nuclear morphology and spatial distributions. The resulting unbiased nuclear representations allow accurate intrinsic tissue properties to be extracted computationally. This avoids artifacts associated with cycle consistency in bidirectional transformation.

Furthermore, the NOCH images generated from the tri‐modal combination and the H&E images displayed no significant differences in the intensity of hematoxylin and eosin components and in the hematoxylin proportion (Figure 4c). Specifically, the hematoxylin proportion for H&E and tri‐modal NOCH are 0.40 (CI = 0.38, 0.42) and 0.41 (CI = 0.40, 0.43), respectively, indicating a remarkable similarity in staining. In contrast, NOCH images generated from other modalities exhibited significant differences, underscoring the large bias in their virtual staining outcomes. We examined the consistency of hematoxylin proportions across various cancer stages and found them to be similar to those observed in H&E staining (Figure S7, Supporting Information). For additional insights, we visualized the nuclear segmentation results of NOCH images in seven different modalities (Figure S8a–h, Supporting Information), along with the total number of nuclei (Figure S8i, Supporting Information). A comprehensive summary of the corresponding numbers, total area, and size of nuclei is provided in Table S1 (Supporting Information). These findings collectively highlight the nuclear characteristics of the tri‐modal NOCH approach, which closely aligns with H&E staining (with an error margin of <2%).

### 2.6 Automatic Cancer Stage Classification

**Figure 5:**
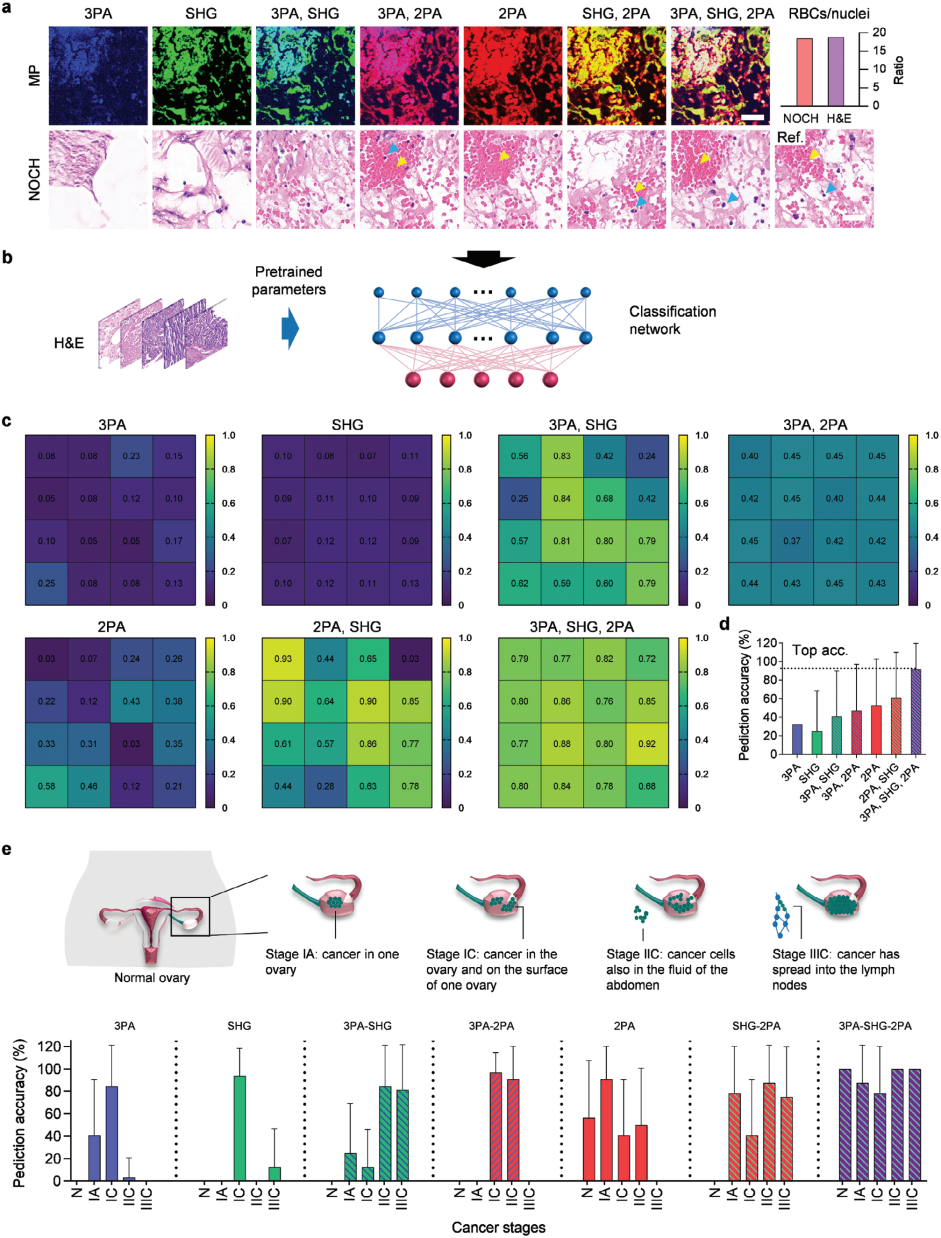
Pathological classification based on the NOCH images. a) NOCH results produced from different modality permutations and combinations. Blue and yellow arrowheads indicate nuclei and red blood cells (RBCs), respectively. Histogram shows RBCs/nuclei ratio of the tri‐modal NOCH and reference. Scale bars, 150 µm. b) Pathological classification using NOCH images with model pretrained on H&E data. c) Predicted probability distribution for an ovarian tissue of cancer Stage IIC. One cell corresponds to an area of 334 × 334 µm2. d) Comparison of total prediction accuracy for all classes (mean ± SD, n = 128). e) Illustration of different ovarian cancer stages (top) and the corresponding prediction accuracies of different modalities (mean ± SD, *n* = 128).

To evaluate the potential of NOCH for computational diagnostics, we conducted cancer stage classification on the virtually stained images using downstream machine learning analyses. First, we trained a classification model, which combines the deep layer aggregation (DLA)^[^ ^27^ ^]^ and Res2Next,^[^ ^28^ ^]^ using the real H&E data (**Figure** **5**a,b). This network achieved the highest prediction accuracy while maintaining model conciseness and efficient running times, outperforming various classification models^[^ ^27^, ^28^, ^29^, ^30^, ^31^, ^32^, ^33^, ^34^, ^35^, ^36^, ^37^, ^38^, ^39^, ^40^, ^41^ ^]^ as summarized in Table S2 (Supporting Information). We utilized this pretrained classification network to evaluate NOCH images for pathological diagnosis. The predicted probability distribution on the NOCH images are depicted in Figure 5c, revealing regional differences in diagnosing ovarian cancer tissues. Notably, the combination of the three NOCH modalities consistently yielded probabilities mostly >0.6, outperforming other modalities. When combining information from these signals to classify ovarian cancer stages, the total prediction accuracy reached >91% (CI = 86.48%, 96.33%), much higher than that of the other modalities (Figure 5d).

For specific cancer classes (Figure 5e), single 3PA, which has a low signal‐to‐noise ratio (SNR),^[^ ^6^, ^42^ ^]^ and single SHG, which mainly provides collagen information, showed low accuracy (below 30%) in most cancer staging. Their limited accuracy stemmed from the lack of differentiation in their NOCH images. In contrast, NOCH images obtained from 2PA demonstrated higher prediction accuracy due to the rich structural information it provided. Combining two modalities yielded significant improvements. However, accurately classifying four or more cancer stages remained challenging for these modalities, except when the three modalities were combined. The combination of the triple NOCH modalities achieved the highest classification accuracy, particularly when distinguishing between normal, cancer IIC, and IIIC tissues, approaching 100% (Figure 5e). This was attributed to the distinct characteristics of the NOCH images of these tissues, including structural morphology and nuclear density, which set them apart from other cancer tissues. The results highlight the advancement of our method and the importance of combining the three MP signals, which consist of crucial information about cancer‐associated collagen (CAC) through SHG and redox‐ratio data via NADH and FAD.^[^ ^6^, ^9^, ^13^ ^]^ Consequently, the contrastive translation results of NOCH images derived from three simultaneously collected MP signals hold significant potential for cancer staging, eliminating the need for cumbersome tissue preparation and staining.

While various style transfer networks, such as pix2pix translation with conditional adversarial networks^[^ ^43^ ^]^ and unpaired translation with a cycle‐consistent loss,^[^ ^25^ ^]^ have been proposed for efficient style transfer, their performance in computational histology remains limited, particularly when dealing with tissues exhibiting complex microstructures and pathological conditions. Cycle‐GAN and pix2pix translation tend to be more susceptible to translation artifacts compared to NOCH. We experimented these models and contrastive learning with alternative generators such as UNet,^[^ ^44^ ^]^ StyleGAN2,^[^ ^45^ ^]^ or 6‐block ResNet^[^ ^25^ ^]^ for cancer staging (Figure S9, Supporting Information). These substitutions resulted in the generation of additional superfluous textures with a lower prediction accuracy. Our comparative studies revealed important distinctions between different learning methods for virtual histological staining and cancer staging. When applied to NOCH images, single modalities like 3PA and SHG showed poor diagnostic accuracy from insufficient differentiation. However, the combination of all three nonlinear modalities enabled accurate multi‐class cancer staging by providing complementary structural, collagen, and metabolic information. Together, these assessments demonstrate the importance of synergizing appropriate learning approaches with information‐rich multi‐parametric imaging to unlock diagnostic capabilities.

## 3 Discussion

The high‐throughput nonlinear signal measurements provide sufficient foundational molecular contrast from unperturbed clinical tissues. This study establishes NOCH as a promising new technique for rapid, non‐invasive histopathology without exogenous stains or dyes. This leverages information‐rich nonlinear datasets to enable histopathology‐like, structure‐mapping, intuitive analysis of the tumor microenvironment. By using contrastive deep learning to convert intrinsic label‐free nonlinear signals into virtual H&E slides, NOCH enables stain‐free pathology for fresh tissues. Our results demonstrate NOCH can generate diagnostically useful and accurate virtual histology across various sample types and cancer stages. Compared to conventional cycle‐consistent networks, contrastive translation produced fewer staining artifacts and higher morphological fidelity. Effective information maximization between corresponding nonlinear and histological patches enabled accurate virtual H&E rendering even from single sample images. Notably, combining all three nonlinear modalities of 3PA NADH, 2PA FAD, and SHG yielded optimal virtual staining and diagnostic classification accuracy. The rich structural, collagen, and metabolic information provided by this tri‐modal approach cannot be matched by any single modality. This highlights the importance of leveraging multiparametric nonlinear data for virtual pathology.

Interestingly, we combined high‐speed galvo‐resonant scanning, image denoising, and computational histology to realize faster cancer diagnosis and analysis workflows (Figure S10, Supporting Information). This pipeline achieved significant speed‐up over the high‐quality dual‐galvo scanning and computational histology pipeline, with a slightly reduced accuracy despite lower photochemical/thermal stress (Note S3, Supporting Information). The optimized pipeline may balance speed versus accuracy, advancing computational staining and characterization of heterogeneous tumor microenvironments.

However, some potential shortcomings and limitations should be considered. First, it is evident that the image datasets employed for training and validation, while substantial for research purposes, are relatively modest in scale. A more diverse dataset will enable the network to encounter a broader spectrum of tissue microstructures, pathological conditions, and variations in staining patterns, thereby potentially improving its diagnostic accuracy and generalizability. Second, it is imperative to recognize that systematic clinician review and validation remain indispensable components of the diagnostic process. While our network has exhibited remarkable potential in generating virtual histopathological images, these results must undergo rigorous clinical validation, particularly in comparison to traditional H&E staining. Third, label‐free nonlinear microscopy techniques have limited imaging depths that may hinder applications for thick or opaque specimens. Biological specimens with considerable depth or those with inherent opacity necessitate complementary imaging modalities for comprehensive tissue assessment. Finally, it is worth noting that the computational complexity of NOCH networks can pose challenges, particularly when striving for real‐time image analysis. Striking a balance between computational efficiency and diagnostic accuracy will be a critical consideration in the practical implementation of NOCH imaging for routine clinical use.

Future research endeavors will explore the applicability of the nonlinear optical modalities in a broader spectrum of tasks, such as Jones satin, Masson's Trichrome stain, and collagen and vesicle^[^ ^11^ ^]^ segmentation. Achieving virtual stain in these areas will necessitate the enhancement of the relatively weak MP signal, specifically 3PA, to mitigate potential noise interference. To this end, employing a laser source with a compressed pulse width on the order of tens of femtoseconds and a low repetition rate of <10 MHz holds promise. Such a laser configuration would efficiently excite the 3PA signal at a bio‐safety energy level while maintaining high intrinsic contrast.^[^ ^13^ ^]^ Consequently, the acquisition of unambiguous, high SNR images can be expected to yield more precise and artifact‐free computational histology.

Furthermore, the development of effective style transfer solutions for virtually staining complex biological structures has become an imperative demand within the biomedical optics community. Addressing this challenge will enhance the versatility of our approach, enabling the virtual staining of a wider array of biological tissues and structures. In addition to these efforts, the construction of more diverse datasets will represent a significant advancement in improving the applicability of the network. This diversification encompasses not only the inclusion of various patient data but also the incorporation of diverse imaging data from other nonlinear signals, such as third‐harmonic generations and fluorescence lifetime. Finally, the ability to accurately discriminate different ovarian cancer subtypes, identify borderline or low malignant potential tumors, and grade morphological features like nuclear pleomorphism could better showcase the capabilities of our nonlinear optical imaging and deep learning workflow. We chose FIGO staging as a first classification target given the well‐defined labels across cancer progression. But expanding the classification tasks to address more subtle pathological complexities is a valuable next step. We hope to systematically assess whether this multidimensional imaging data combined with tailored deep learning classifiers can help unravel diagnostic complexities beyond traditional histopathology. Characterizing challenging cases of mimicry and differential diagnosis using this computational approach could better reveal its potential.

In conclusion, the interdisciplinary intelligent method we have introduced represents a significant advance in biomedical‐related computer vision tasks. Its demonstrated superiority over conventional methods in clinical diagnosis and postoperative analysis positions it as a valuable tool with the potential to impact multiple domains within biology and medicine.^[^ ^46^, ^47^ ^]^ As we continue to refine and expand upon this methodology, we anticipate further breakthroughs and applications that will benefit both researchers and clinicians alike.

## 4 Experimental Section

### Optical Setups

The nondescanned ultrafast imaging microscope mainly includes the excitation path and emission path. The excitation path mainly consists of a femtosecond laser, a hybrid scanner with scan lens and tube lens for high‐ and slow‐speed scanning, and an objective. The ultrafast femtosecond laser (pulse width: ∼100 fs, repetition rate: 80 MHz, Chameleon Discovery, Coherent) was compensated with an 8000‐fs2 group delay dispersion (GDD). This GDD minimized photochemical and thermal stress and avoided photodamage and image distortion, and hence allowed a more efficient 1140‐nm excitation under bio‐safety level.^[^ ^9^, ^13^, ^42^ ^]^ An autoalignment system could collimate the laser beam rapidly when the motorized mirrors switch the beam between the GRS for high‐speed acquisition and DGS for high‐quality imaging.

The emission path mainly includes an IR‐cut filter (ET750sp‐2p8, OD8, Chroma) for blocking the backscattered laser, a DM for separation of the excitation light and emission signals, and nondescanned detectors with DMs and bandpass filters combination for detecting different modality nonlinear signals. The longpass of 488 nm and bandpass of 450/50 nm, longpass of 593 nm and bandpass of 570/10 nm, and longpass of 685 nm, and bandpass of 641/75 nm filters spectrally separated the trichromatic channels of 3PA NADH, SHG (with incidental 3PA FAD), and 2PA FAD, respectively. The crosstalk of 3PA FAD in SHG was filtered out by performing *I*_SHG_ − *I*_2PA_ since 2PA FAD and 3PA FAD share the same morphology. The scan field of view (FOV) of a single image was ≈634 µm × 634 µm with the apochromatic objective (MRD70200, 20×, 0.75 NA, Nikon), while the large images were formed with multiple scan FOVs and stitched by blending with 50% overlap to minimize the splicing trace. The acquisition speed of a MP image consisting of three modalities at the dual‐galvo scanning mode was 0.474 Hz with a pixel dwell time of 2 µs (1024 × 1024 pixels), which was the speed usually applied to obtain a high‐SNR image,^[^ ^42^ ^]^ while the acquisition speed at the galvo‐resonant scanning mode was maximally 30 Hz with a pixel dwell time of 0.5 µs (256 × 256 pixels).

### Sample Preparation

Ovary, breast, and liver tissues were collected from patients at China‐Japan Union Hospital of Jilin University, Shaanxi Provincial Cancer Hospital, and Shenzhen University General Hospital, respectively, with approval of Medical Ethics Committee, Shenzhen University Medical School (PN‐202300128). Physicians and surgeons recruited patients and obtained study consent. Prospective enrollment began on June 1, 2019, and closed on June 1, 2023. The study used de‐identified leftover tissue specimens that were archived. Surgically removed tissue samples were snap‐frozen in liquid nitrogen and stored at −80 °C until being cut into 5‐µm sections for unstained and stained applications using a freezing microtome (CM1850, Leica, Germany). Unstained and stained sections were continuous in depth. The frozen tissue sections were simply covered with a coverslip, imaged by MP microscopy, and stored at −80 °C. The adjacent slices were processed with standard H&E staining including fixing tissue slide in acetic acid, staining with hematoxylin and eosin mixture, dehydrating with ethanol, cleaning with xylene, etc. The H&E images for reference were taken by a bright‐field microscope. Two gynecological oncologists with experience in ovarian cancer performed cancer stage identification and pathological analysis on the histological sections according to the International Federation of Gynecology and Obstetrics (FIGO) classification standards.

### Network Architecture

For the asymmetric image transformation, the concept of cycle‐consistent loss takes precedence (e.g., CycleGAN,^[^ ^25^ ^]^ UNIT, MUNIT, and MuGAN^[^ ^48^, ^49^, ^50^ ^]^). These approaches involved learning bidirectional mapping between the input and target domains. Nevertheless, this loss framework heavily relies on the strict assumption of a bijective relationship between the input and target domains. When one domain's image contains more information than the other, achieving satisfactory results becomes considerably more challenging.

In order to avoid the bijection limitation imposed by cycle‐consistent learning, contrastive learning was used to maximize mutual information that the relations present in the input image should also be similarly present in the resulting image. Before that, the study defined the input domain of set $X\subset R^{H\times W\times C}$, which can be translated to the output domain of set $\hat{Y}\subset R^{H\times W\times C^{\prime }}$, approaching the target domain of set $Y\subset R^{H\times W\times C^{\prime }}$, where *H* and *W* are the height and width of the image, respectively. *C* represents the input nonlinear modalities. *C*′ represents the output channels corresponding to hematoxylin and eosin for NOCH. The datasets are given as paired instances X={x∈X} and Y={y∈Y}.

The generator *G* of the network architecture for NOCH consists of three parts: the encoder *G*_enc_, the transformer *G*_tran_, and the decoder *G*_dec_, which were applied sequentially to produce output image of

(1) $$\hat{y}=\;G\;\left(x\right)=G_{dec}\;\left(G_{tran}\left(G_{enc}\left(x\right)\right)\right)$$

The encoder *G*_enc_ consists of three convolutional layers to extract features from the input image, which compresses the image into 256 64 × 64 feature vectors. The *G*_tran_ combines the dissimilar features of the image, converting the feature vectors of the image in the input domain into the feature vectors in the output domain. The study used a nine‐layer ResNet module,^[^ ^25^ ^]^ each of which composes of two convolutional layers, which could preserve the original image features during transformation. The decoder *G*_dec_ consists of two deconvolutional layers and subsequent one convolutional layer to restore the low‐level features from the feature vectors, and finally the generates the images.

The mutual information between the input and generated images was maximized by the noise contrastive estimation framework which was used to transform the multi‐classification problem into a bi‐classification problem.^[^ ^23^ ^]^ The multilayer perceptron combined with the encoder extract the positive feature sample in the patches of the NLOI images, which matches the query from the output image, and the negative feature samples in other patches of the same NLOI image (self‐contrastive) or of other NLOI images (cross‐contrastive). The query, positive, and negative samples are respectively mapped to *k*‐dimensional vectors:

(2) $$\upsilon ,\;\;\upsilon^{+}\in R^{K},\;\upsilon^{-}\in R^{N\times K}$$

where N = 255 is the number of negatives (sufficient negatives yield better performance in practice) and $\upsilon_{n}^{-}\in R^{K}$ is the *n*th negative. The vectors are normalized to a unit sphere to prevent spatial expansion or collapse. This thereby forms a classification problem of N + 1 way. The probability of the positive example being selected over negatives is obtained by calculating the cross‐entropy loss,

(3) $$l\;\left(\upsilon ,\;\upsilon^{+},\upsilon^{-}\right)=\;-\log\left(\frac{\exp\left(\upsilon \cdot \upsilon^{+}/\tau \right)}{\exp\left(\upsilon \cdot \upsilon^{+}/\tau \right)+\sum_{n\;=\;1}^{N}\exp\left(\upsilon \cdot \upsilon_{n}^{-}/\tau \right)}\right)$$

where τ is the proportional hyperparameter, often referred to as the temperature coefficient, scaling the distances between the query and other examples. A large τ would lead to a smooth distribution of the logits, i.e., $\upsilon \cdot \upsilon^{+}$, and hence the loss would treat all negative samples equally, resulting in no weight of model learning. A minute τ would lead to a sharp distribution of the logits, and hence the model would pay more attention to the particularly difficult negative samples, which in fact, could be potential positive samples. This may result in the converging difficulty or poor generalization ability of the model. In this work, τ is set to be 0.07. The cross‐entropy loss can be used to calculate the contrastive losses.

For image style transfer tasks that focus on pixel alteration, the information of each pixel of an image sample is very important. Thus, the batch normalization that considers the content of all images in a batch when calculating normalization statistics, which leads to inattention of the unique details of each sample, was inappropriate in this work. Similarly, algorithms such as layer normalization that considers all channels of a sample may ignore the differences between different channels, and hence are not suitable. Instance normalization that was more suitable for scenes with higher requirements for a single pixel was appropriate for style transfer applications. All elements of a single sample and a single channel were considered when the instance normalization calculates the normalization statistics. In practice, using batch normalization with learnable affine parameters instead of instance normalization for the generator and discriminator did not provide better results.

Contrastive patch‐wise learning offers the unique ability to extract negative image blocks from a single image, eliminating the need for multiple images in the dataset. This allows for the achievement of computational histology on a single image (Figure 3a–c).

### Network Loss Functions

The network utilizes the adversarial loss and the contrastive loss to construct a loss function. The adversarial loss ensures the generated image to visually approaches the image from the target domain during the generative adversarial imitation learning, which can be expressed as

(4) $$L_{GAN}\;\left(G,D,X,Y\right)=E_{y∼Y}\;\log D\left(y\right)+E_{x∼X}\log\left(1-D\left(G\left(x\right)\right)\right)$$

where *G* is the generator consisting of the encoder *G*_enc_, the transformer *G*_tran_, and the decoder *G*_dec_. D is the Markovian discriminator known as patch‐GAN, *E* is the mathematical expectation.

The feature layers of the input image were extracted by the encoder *G*_enc_, in which different layers and different spatial positions represent different image patches. The deeper the feature layers correspond to the larger image patches. *L* layers of interest and passed these feature maps were selected through a small two‐layer multilayer perceptron (MLP) network, *H_l_* following *G*_enc_, which produce a stack of features

(5) $${\left\{z_{l}\right\}}_{L}={\left\{H_{l}\left(G_{enc}^{l}\left(x\right)\right)\right\}}_{L}\;$$

where $G_{\mathrm{enc}}^{l}$ is the output of the *l*‐th chosen layer (*l* ∈ {1, 2, …, *L*}). Similarly, the feature stack of the output image of $\hat{y}$ can be extracted as

(6) $${\left\{{\hat{z}}_{l}\right\}}_{L}={\left\{H_{l}\left(G_{enc}^{l}\left(G\left(x\right)\right)\right)\right\}}_{L}\;$$

The study denoted *s* ∈ {1, 2, …, *S_l_*}, where *S_l_* is the number of spatial locations in each layer. Then, the corresponding positive feature can be referred as $z_{l}^{s}\in R^{C_{l}}$ and other features as $z_{l}^{S\setminus s}\in R^{\left(S_{l}-1\right)\times C_{l}}$, where *C_l_* is the feature dimension (channel number). The contrastive loss can be calculated by matching the corresponding input‐output patches at a specific location, while the patches at other positions of the same image are taken as negatives:

(7) $$L_{contrast}\;\left(G,H,X\right)=E_{x∼X}\;\sum_{l\;=\;1}^{L}\sum_{s\;=\;1}^{S_{l}}l\left({\hat{z}}_{l}^{s},z_{l}^{s},z_{l}^{S\setminus s}\right)$$

where l represents the cross‐entropy loss for the N + 1 way classification.

Likewise, the contrastive loss for the $\hat{y}$‐domain version (taking *x* as reference), which is similar to the consistent loss, can be written as

(8) $$L_{contrast}\;\left(G,H,Y\right)=E_{y∼Y}\;\sum_{l\;=\;1}^{L}\sum_{s\;=\;1}^{S_{l}}l\left(z_{l}^{s},{\hat{z}}_{l}^{s},{\hat{z}}_{l}^{S\setminus s}\right)$$

Therefore, the total loss function can be expressed as

(9) $$L_{total}=\;\lambda L_{GAN}\left(G,D,X,Y\right)+\lambda_{X}L_{contrast}\left(G,H,X\right)+\lambda_{Y}L_{contrast}\left(G,H,Y\right)$$

The weight of the adversarial learning loss λ  =  1 and the weight of the contrastive losses λ_*X*_ =  1 and λ_*Y*_ =  1 were selected for superior performance in the translation.

### Training Options

The neural network was trained using data from NLOI and bright‐field H&E imaging. The large‐field images were split into 340 × 340 pixels for SRS NOCH and 544 × 544 pixels for MP NOCH to mitigate the storage requirement of the GPU. The testing and training had a split ratio of 1:6 for the SRS dataset and 1:4 for the MP dataset. The contrastive learning framework employs random on‐the‐fly patch extraction and augmentation during training. The framework frequently examines random validation patches extracted from the train set to assess translation fidelity during training. The network was effectively trained until it translated nonlinear optical validation patches into realistic histological counterparts closely matching the target morphology. The epoch with optimal qualitative validation set performance was selected as the final model before testing on the held‐out test data. Paired training helps match pathological features between domains given the high tissue complexity across cancer stages. This validation approach assesses generalization similar to conventional validation but was naturally integrated into the contrastive learning framework.

The weight of the adversarial learning loss was λ = 1. The weight of the self‐contrastive losses were λ_*X*_ = 1 and λ_*Y*_ = 1. The adaptive moment estimation of Adam with momentum terms of β_1_ = 0.5 and β_2_ = 0.999 was used as the optimizer of the generator and discriminator. The initial learning rate for Adam was 0.0002.

### Benchmarks

For label‐free computational histology, the pix2pix translation^[^ ^43^ ^]^ and Cycle‐GAN^[^ ^25^ ^]^ with the same residual layers in the generator and the same patches in the discriminator were optimized to demonstrate the competitive performance. The Cycle‐GAN exhibited an ≈0.75 slow‐down compared to the contrastive network in the network training of 245760 iterations. The contrastive learning model was also tested with various generators such as UNet,^[^ ^44^ ^]^ StyleGAN2,^[^ ^45^ ^]^ or 6‐block ResNet^[^ ^25^ ^]^ for cancer staging.

For automatic pathological classification, typical networks with pretrained weights were selected with superior accuracy in distinguishing cancer stages using the H&E histopathological dataset compared to their own variants with different model sizes. All classification models were trained in exceeding 60 epochs for selection of the best checkpoints with the highest accuracy. The NOCH images of different MP modality combinations were tested for automatic pathological classification using the pretrained best model.

### Data Processing

Since the background of MP images (black) was opposite to the that of bright‐field histopathological images (white), the color channels of the NLOI image were inverted by *I_input_* = |*L* − *I*_raw_|  to avoid content mismatching during image splitting, where *L* is the dynamic range of the image (e.g., 255 for 8‐bit). This method, rather than adding additional constraints of background pixel intensity into the network, avoids model complexity.

The classification network predicted the probability of normal tissue and cancer stage IA, IC, IIC, and IIIC. The accuracy on the classification error map was a δ‐function which can be defined as

(10) $$Acc\;=\left\{\begin{array}{c} 1,\;Class\left(prob_{max}\right)=Class_{ground-truth}\\ 0,\;Class\left(prob_{max}\right)\neq Class_{ground-truth} \end{array}\right.\;$$

where *Class*(*prob*_max_) is the inferred class with a maximum probability (*prob*_max_) among the pathological classes.

The intensity of hematoxylin component was measures as the mean value of its deconvolution color‐inverted images ( *I_hemat_* = |*L* − *I*_deconv_| ). The hematoxylin proportion in the stained images are calculated as

(11) $$\mathrm{Prop}\;=\frac{I_{hemat}}{I_{hemat}+I_{eosin}}\;$$

where *I_eosin_* is the intensity of eosin component.

### Statistical Analysis

Sample sizes and statistical analyses including the mean, SD, and significant difference were specified in figure legends and text for each experiment. The black dashed lines and black solid lines in the violin plot represent the quartile and median positions, respectively. The black solid lines in the scatter plot indicate the mean. All CIs were constructed at a 95% confidence level. The statistical analyses of the mean, SD, CI, and significant difference were performed using GraphPad Prism and MATLAB.

## Conflict of Interest

The authors declare no conflict of interest.

## Author Contributions

B.S. modified the network and conducted the deep learning. Z.L. and B.S. performed the multimodal imaging and prepared the datasets. Y.P., Y.G., and Z.Y. processed the tissues and analyzed the pathological states and image data. R.H., L.L., and J.Q. supervised the data analysis. L.L. and J.Q. supervised the project, obtained funding, and edited the manuscript. All authors contributed to writing the manuscript.

## Supporting information

Supporting Information

## Data Availability

The main data supporting the findings of this study are available within the paper and its Supplementary Information. The training and testing data for SRS to H&E task are publicly available at https://doi.org/10.7910/DVN/EZW4EK. The training and testing data for MP to H&E task will be made publicly available at https://github.com/shenblin/NOCH/blob/main/datasets/README.md. All data used in this study are available from the corresponding author upon reasonable request.
