# Supplementary material for: Noninvasive Nonlinear Optical Computational Histology
Source: Adv Sci (Weinh). 2023 Dec 14;11(9):2308630. doi: 10.1002/advs.202308630 (PMC10916666; doi:10.1002/advs.202308630)
Supplement: Supplementary file 1 — Supporting Information [file ADVS-11-2308630-s001.pdf]

ADVANCED SCIENCE
Open Access

Supporting Information

for *Adv. Sci.*, DOI 10.1002/advs.202308630

Noninvasive Nonlinear Optical Computational Histology

*Binglin Shen, Zhenglin Li, Ying Pan, Yuan Guo, Zongyi Yin, Rui Hu, Junle Qu and Liwei Liu**

Supplementary Information

Noninvasive Nonlinear Optical Computational Histology

Binglin Shen[1], Zhenglin Li[1], Ying Pan[2], Yuan Guo[3], Zongyi Yin[4], Rui Hu[1], Junle Qu[1], Liwei Liu[1*]

[1]*Key Laboratory of Optoelectronic Devices and Systems of Guangdong Province and Ministry of Education, College of Physics and Optoelectronic Engineering, Shenzhen University, Shenzhen 518060, China.*

[2]*China–Japan Union Hospital of Jilin University, Changchun, 130033, China.*

[3]*Shaanxi Provincial Cancer Hospital, Xi'an, 710065, China.*

[4]*Shenzhen University General Hospital, Shenzhen 518055, China.*

Contents

Supplementary Information 1

Note S1 The key module of Cycle-GAN network 3

Note S2 Transfer learning to breast and liver cancers 4

Note S3 Denoising-enabled faster pathological diagnosis 5

Fig. S1 Comparison of NOCH results of different networks 7

Fig. S2 Large-field virtual stained results of autopsy normal samples. 8

Fig. S3 Comparison of NOCH performance with different SRS modalities. 9

Fig. S4 Virtual stained nuclei of autopsy normal samples 10

Fig. S5 Comparison of the nuclear characteristics 11

Fig. S6 Histopathological transfer learning. 12

Fig. S7 Comparison of NOCH performance with different modalities. 13

Fig. S8 Nuclear segmentation of different NOCH modalities. 14

Fig. S9 Comparison of prediction accuracy of different models. 15

Fig. S10 High-speed acquisition and image denoising enables faster pathological diagnosis. ... 16

Table S1 Nuclear characteristics for Stage IIIC. 17

Table S2 Size, speed and accuracy of different classification models. 18

Supplementary references 19

Note S1 The key module of Cycle-GAN network

The Cycle-GAN model[1-3] (Fig. S1b) transfers the input image x from the domain X to the output image y' in domain Y via the forward generator $G(x)$. Then the newly generated image y' is transferred back to domain X as the image x' via the backward generator $F(y')$. The first consistency loss is to ensure $x \approx x'$. Similarly, the reference target image y can be transferred from the domain Y to the fake image x^* in domain X via the backward generator $F(y)$. Then this image x^* is transferred back to domain Y as the image y^* via the forward generator $G(x^*)$. The second consistency loss is to ensure $y \approx y^*$. The two similarities define a meaningful mapping that does not exist in the paired dataset.

Note S2 Transfer learning to breast and liver cancers

We demonstrated the transfer learning capability of NOCH by applying it to the conversion of other pathological tissues to match their corresponding H&E images (Fig. S6). We employed self-contrastive learning to translate large-field MP images of unprocessed breast tissue obtained through aspiration biopsy into histopathological morphology, utilizing the pretrained NOCH model developed for ovarian tissue. As depicted in Fig. S6a–c, the network-inferred histopathological states closely aligned with ground truth observations, despite variations in the nuclear positions between the NOCH and H&E images. This misalignment was because we examined adjacent sections obtained through continuous cutting. Notably, the fibrous structure identified by SHG and the dense connective tissue indicated by 2PA FAD in breast tissue were faithfully translated into virtual histopathological morphology at the network output, exhibiting a high degree of concordance with H&E staining.

Expanding beyond breast tissue, we also applied the network to intraoperative liver tissue (Fig. S6d–g) without necessitating retraining. Following this learning process, MP images of hepatocytes were rapidly transformed into histopathological morphology. Notably, the cancer-related features (Fig. S6f) closely resembled their counterparts in H&E staining images (Fig. S6g). These results unequivocally establish the network's transfer learning ability.

Note S3 Denoising-enabled faster pathological diagnosis

To further realize a faster workflow for cancer diagnosis and analysis, as well as reduce stored detrimental heats in the samples, we applied contrastive learning in image denoising (Fig. S10a). The network improved the image quality of 30-kHz galvo-resonant scanning (GRS) to compete with 0.47-Hz dual-galvo scanning (DGS), which enables higher speed image acquisition. Despite the indistinct 3PA morphology of the ovarian tissues, after learning, the network largely suppressed the background and noise of the input GRS images and reconstructed clear texture (Fig. S10b) in a negligible time. The acquisition time of the GRS image (33 ms) and inference time of the network (50 ms) were much shorter compared to the DGS acquisition time (2,100 ms), which suggests that the network allows an approximately 24-fold speed-up for the slow MP imaging (Fig. S10c). Nevertheless, eventual clinical translation needs to consider practical workflow integration and costs compared to routine H&E histopathology.

The raw GRS images and reconstructed output images were compared in Fig. S10d, which demonstrates that the noisy fluctuations in the intensity profile of the GRS images, especially the 3PA NADH image with a higher noise level, were greatly reduced. The peak signal-to-noise ratio (PSNR) on average across more than 100 high-noise images exhibit an increase of approximately 7.6 dB for 3PA NADH, 5.3 dB for SHG (with 3PA FAD crosstalk[4]), and 3.0 dB for 2PA FAD (Fig. S10e), which verifies the capability of the network in image denoising.

With the image denoising, scanning fringe artifacts (SFA) resulting from the coupling of the scan and leakage of ambient light) were also removed (Fig. S10f). We compared computational histology performance using the raw GRS images, network enhanced-GRS images, and DGS images as shown in Fig. S10g. The resulting NOCH image translated from the inferior GRS image has much obvious HTA, yet the denoising network conduces to correction of these mistakes. The histopathological morphology produced from the network enhanced-GRS image ($G_{\mathrm{ID}} + G_{\mathrm{HT}}$) might even possibly have less HTA compared to the NOCH result of the DGS image in some cases. We used colour deconvolution to reveal the composition of hematoxylin and eosin in the NOCH images (donut insets in Fig. S10g) and histologically stained image (donut inset in Fig. S10h). The hematoxylin-eosin proportion of NOCH image of the enhanced-GRS (0.69) and DGS (0.77) were closer to that of the ground truth (0.71), while the proportion obtained from the original raw GRS (1.30) had a large bias

toward hematoxylin that revealed a false translation.

We then performed pathological classification using the output NOCH images and the pretrained classification model. The class prediction accuracies (Fig. S10i) indicate that the inferior NOCH image of GRS was prone to misdiagnosis in cancer stages with a low class accuracy of 54%, which could be improved to 84% after the denoising, approaching the accuracy attained by NOCH of DGS. Thus, the network denoising largely reduced the classification errors for the NOCH images of GRS. A tradeoff exists between information acquisition speed (including network inference) and image quality (associated with the prediction accuracy), while the combination of the fast scanning and image denoising provides a possibility to mitigate this conditionality.

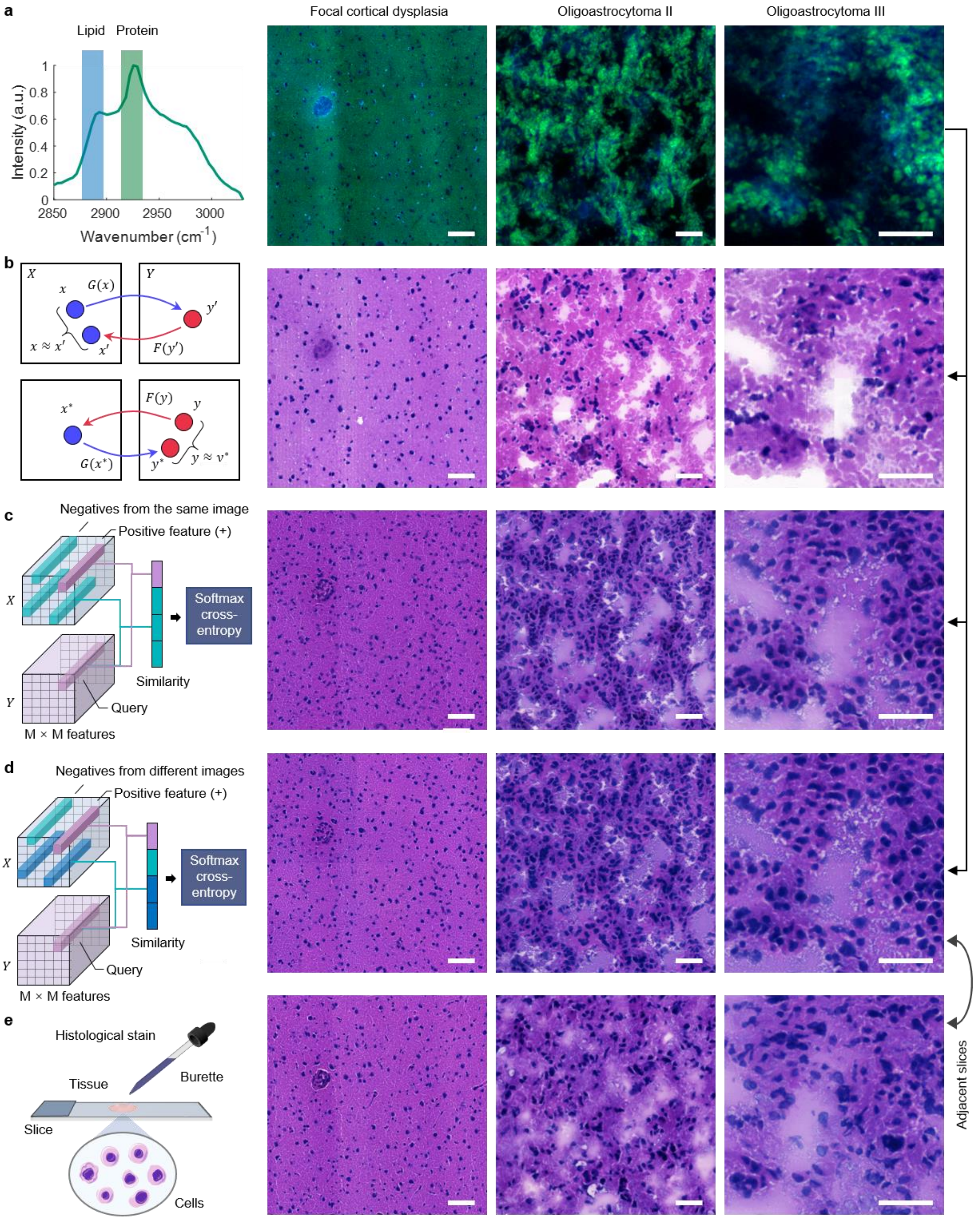

Fig. S1 Comparison of NOCH results of different networks.

a, The SRS spectrum of lipid (blue) and protein (green), which compose the SRS images. **b**, Cycle-consistent module which transforms images between the two domains. **c**, Self-contrastive loss associating the sampled query and its positive, in contrast to negatives within the same image. **d**, Cross-contrastive loss associating the sampled query and its positive, in contrast to negatives

from different images. Second to fourth columns present the virtually histological results. **e**, Conventional H&E histology including fixing tissue slide in acetic acid, staining with hematoxylin and eosin mixture, dehydrating with ethanol, and cleaning with xylene. Second to fourth columns correspond to the bright-field H&E images for reference. Scale bar, 50 μm.

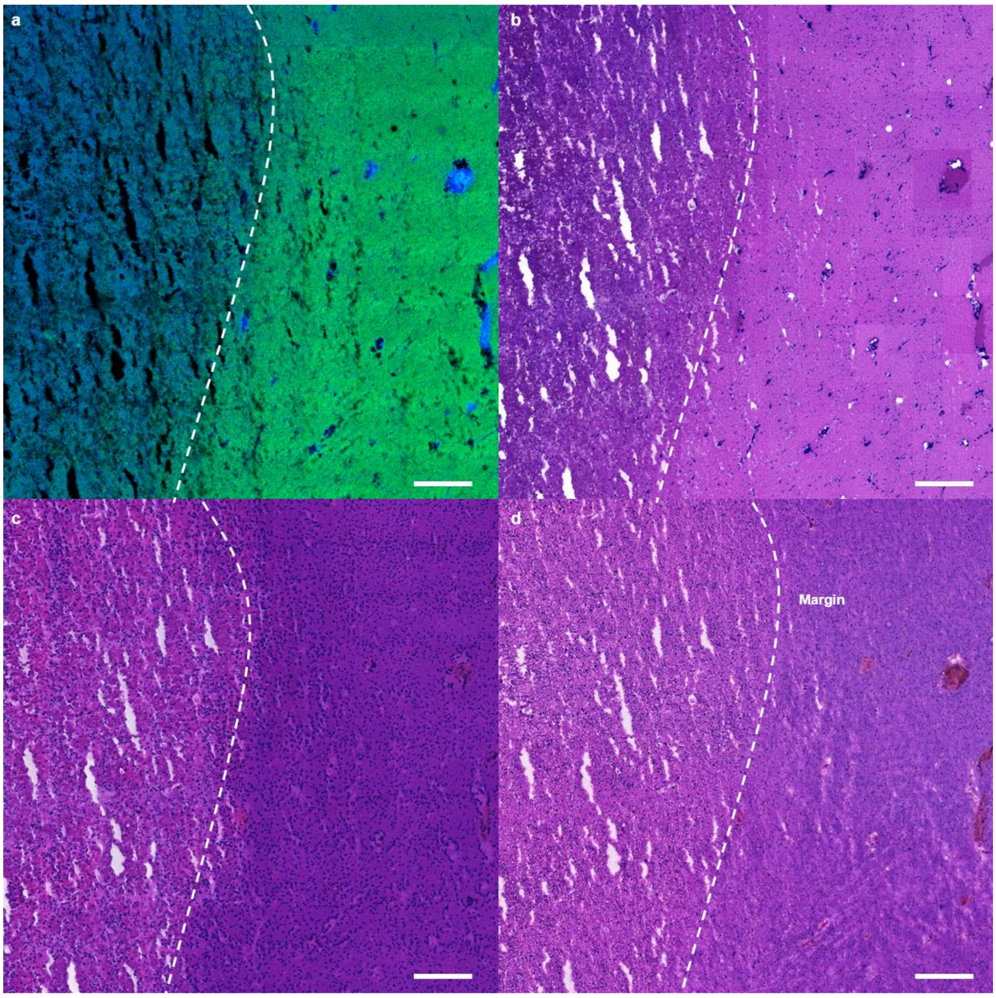

Fig. S2 Large-field virtual stained results of autopsy normal samples.

a, SRS image. **b**, Virtual histological image by Cycle-GAN. **c**, NOCH image by self-contrastive learning. **d**, H&E histological image. White dashed lines indicate the margin. Cycle-GAN generated an inverted staining result compared to H&E, while contrast learning generated a contrast-enhanced histological slice. Scale bars, 400 μm.

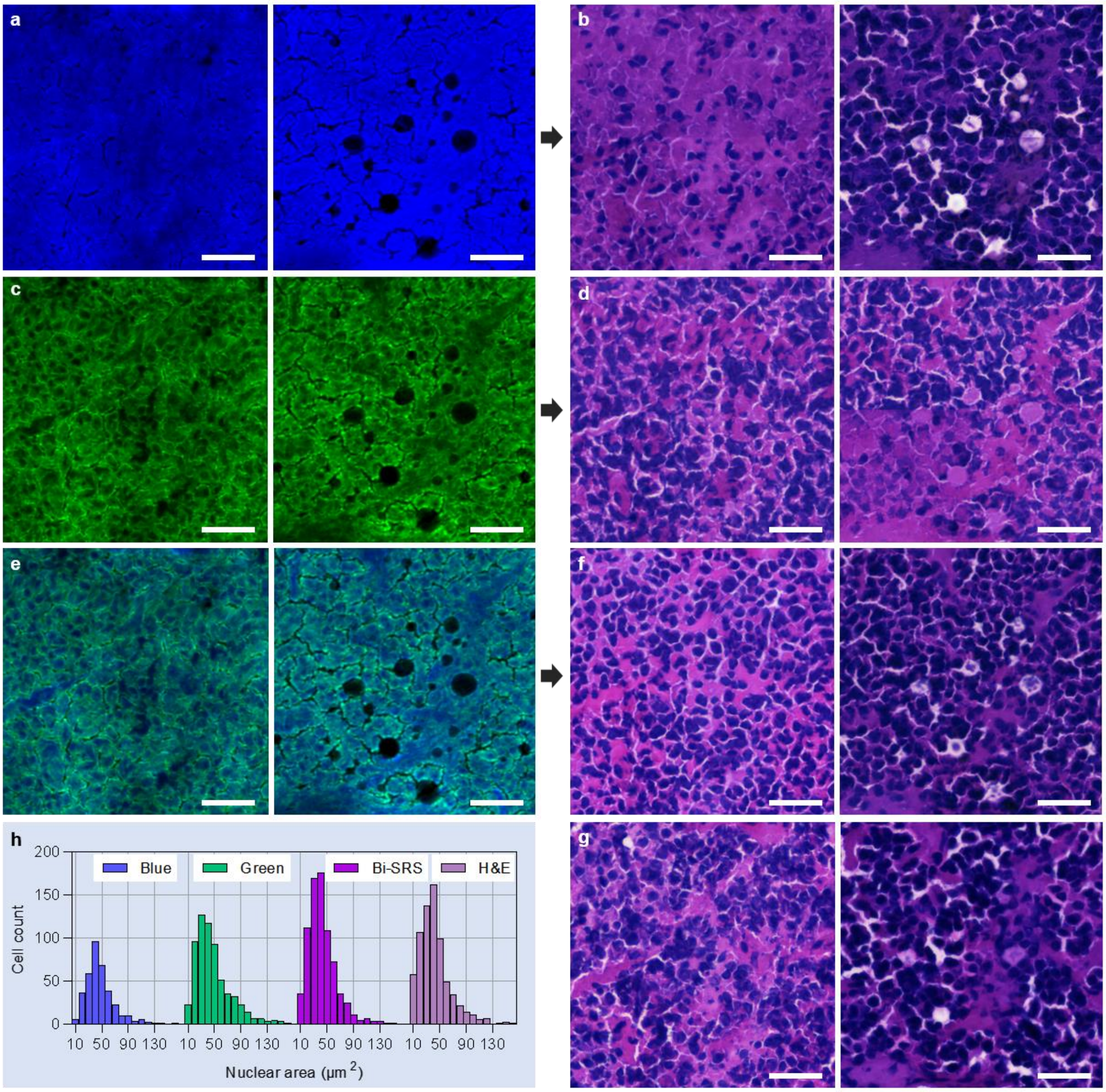

Fig. S3 Comparison of NOCH performance with different SRS modalities.

a, SRS images (blue) at around 2845 cm^{-1}. **b**, NOCH images of **a**. **c**, SRS images (green) at around 2930 cm^{-1}. **d**, NOCH images of **c**. **e**, SRS bimodal images merged by blue and green. **f**, NOCH images of **e** using self-contrastive learning. **g**, H&E histological images. **h**, Histogram distribution of nuclear cross-sectional areas. NOCH of SRS bimodal images can achieve higher staining accuracy across different tissues compared to single modality. Scale bars, 40 μm.

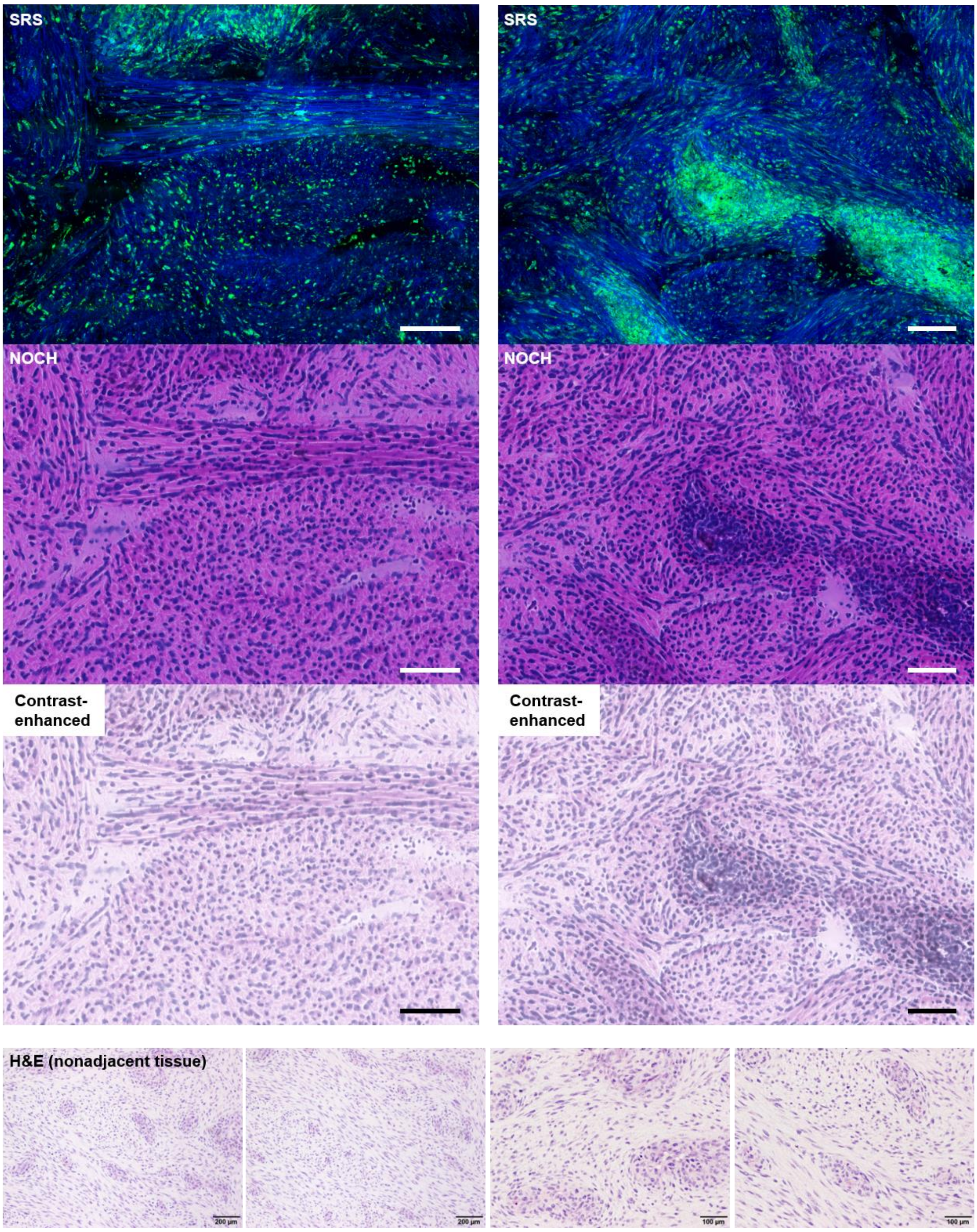

Fig. S4 Virtual stained nuclei of autopsy normal samples.

Recurrent/residual glioblastoma IV. The nuclear contrast-enhanced images parallel with the nonadjacent histologically stained slices. Scale bars, 150 μm.

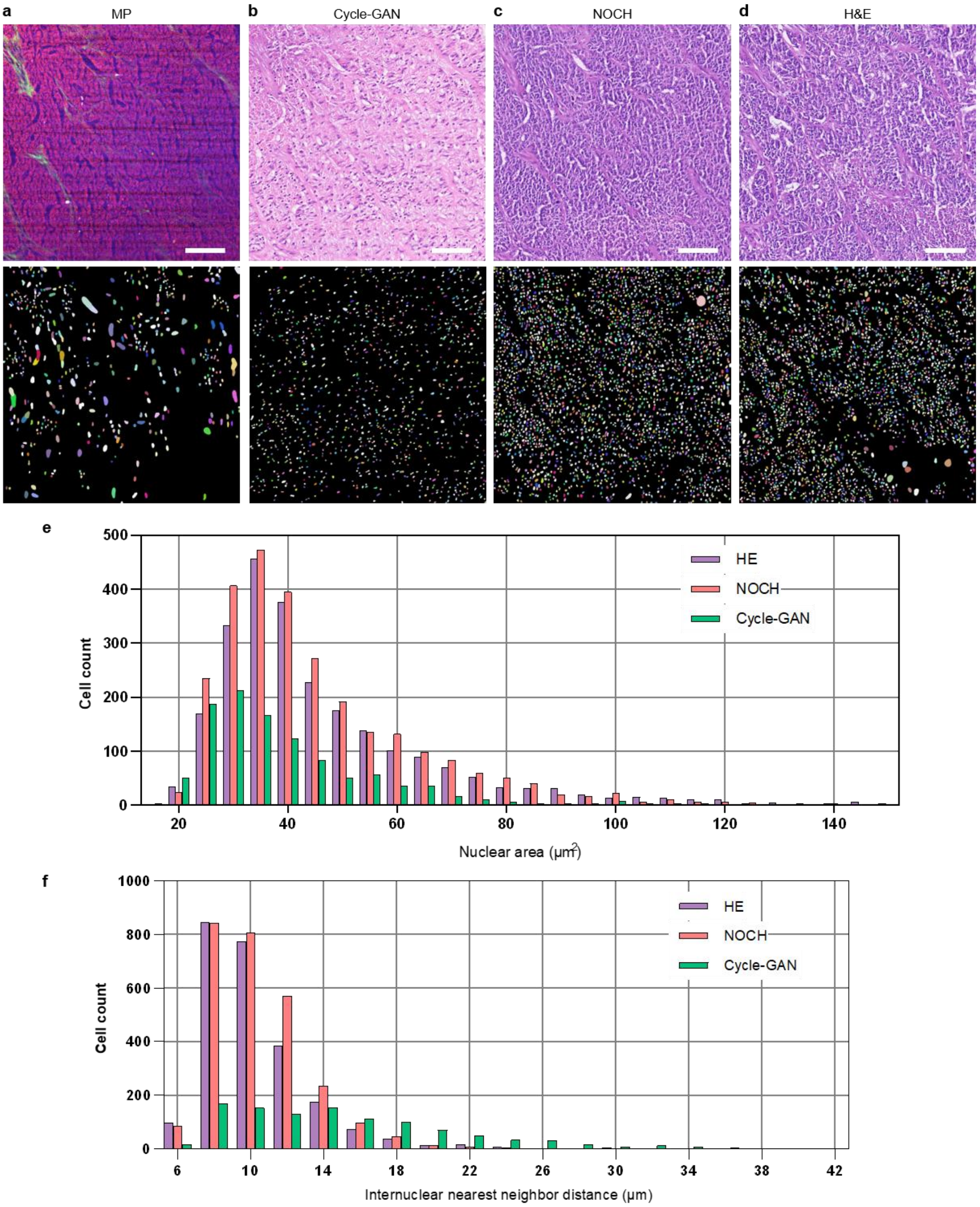

Fig. S5 Comparison of the nuclear characteristics.

MP image (**a**), virtually histological image by Cycle-GAN (**b**) and self-contrastive learning (**c**), and real H&E image (**d**) of Stage IC are presented in the top panel, while the corresponding nuclear extractions are shown in the bottom panel. The nuclei were extracted using StarDist[5]. **e**, Histogram distribution of nuclear cross-sectional areas. **f**, Histogram distribution of internuclear nearest neighbor distances. Scale bars, 150 μm.

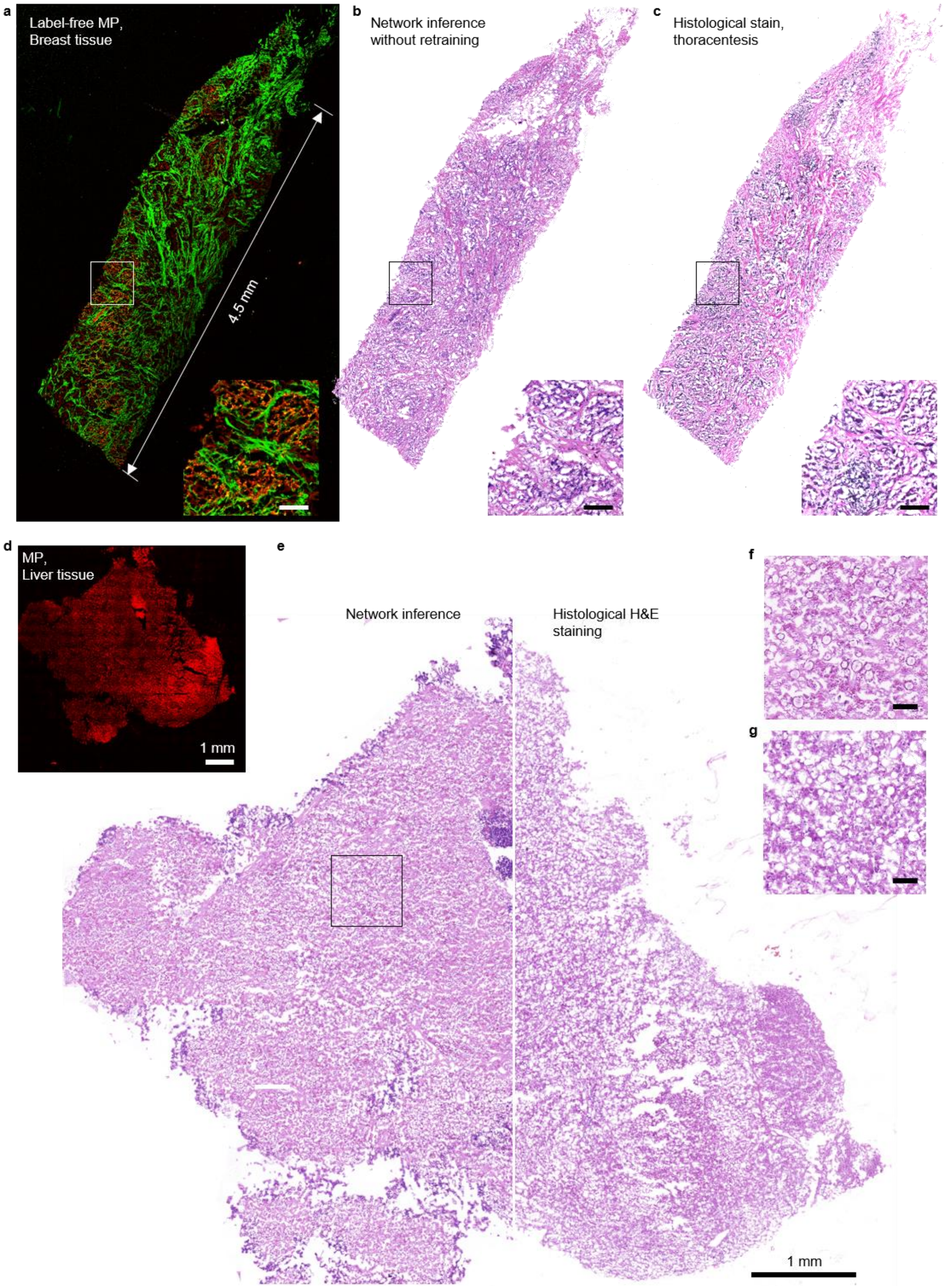

Fig. S6 Histopathological transfer learning.

Large-field MP (**a**), NOCH (**b**), and H&E (**c**) images of human breast cancer samples. **d**, MP image of human liver cancer samples. **e**, The corresponding NOCH image (left) and the adjacent H&E (right) image. Black boxes indicate the zoom-in views magnified in **f** for NOCH and **g** for H&E. The NOCH images were obtained using the pretrained model for ovary. Scale bar: 100 μm for the insets in **a**–**c** and 100 μm in **f**,**g**.

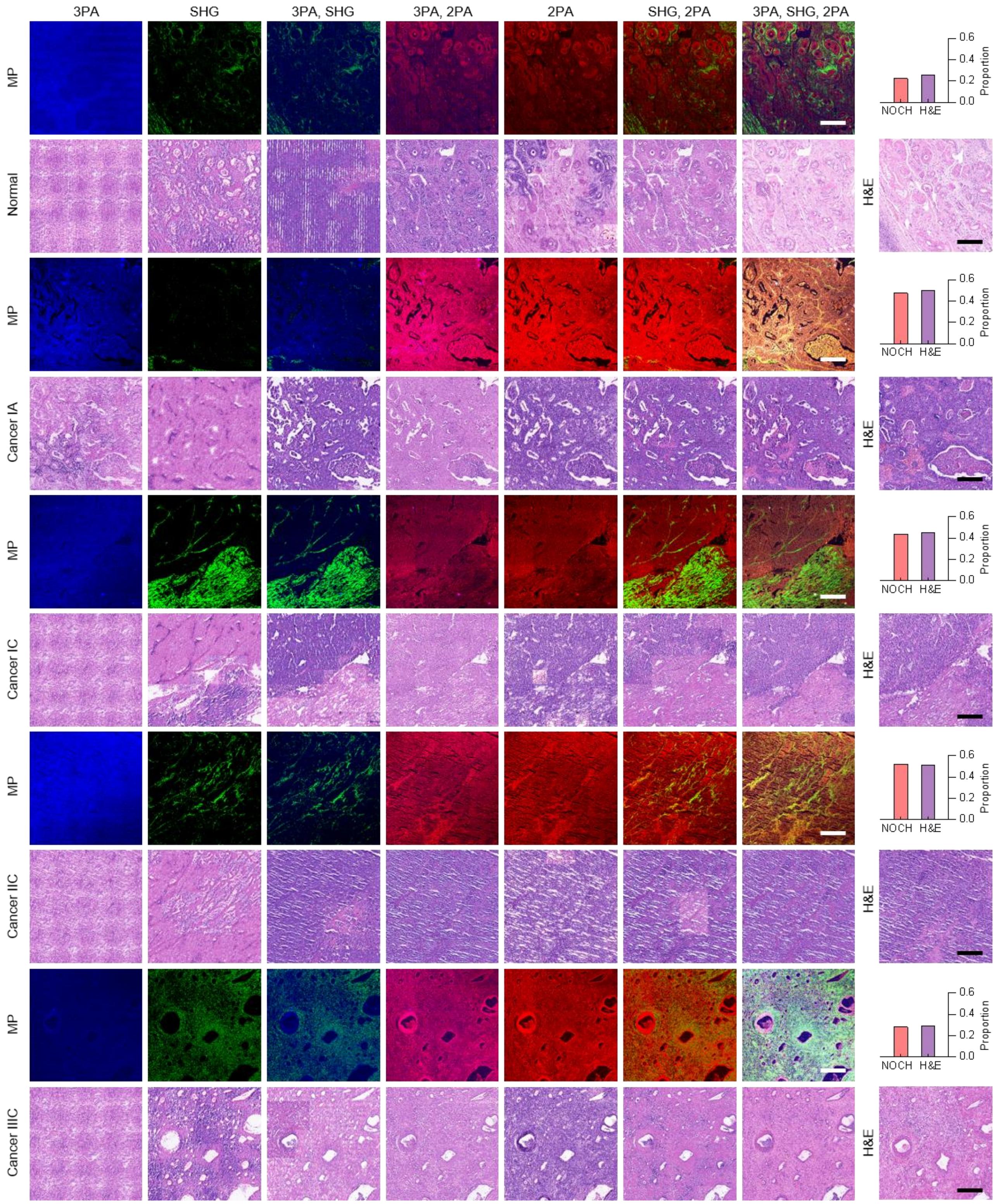

Fig. S7 Comparison of NOCH performance with different modalities.

The histograms in the last column present the hematoxylin proportion of the tri-modal NOCH and reference for each cancer stage. Scale bars, 200 μm.

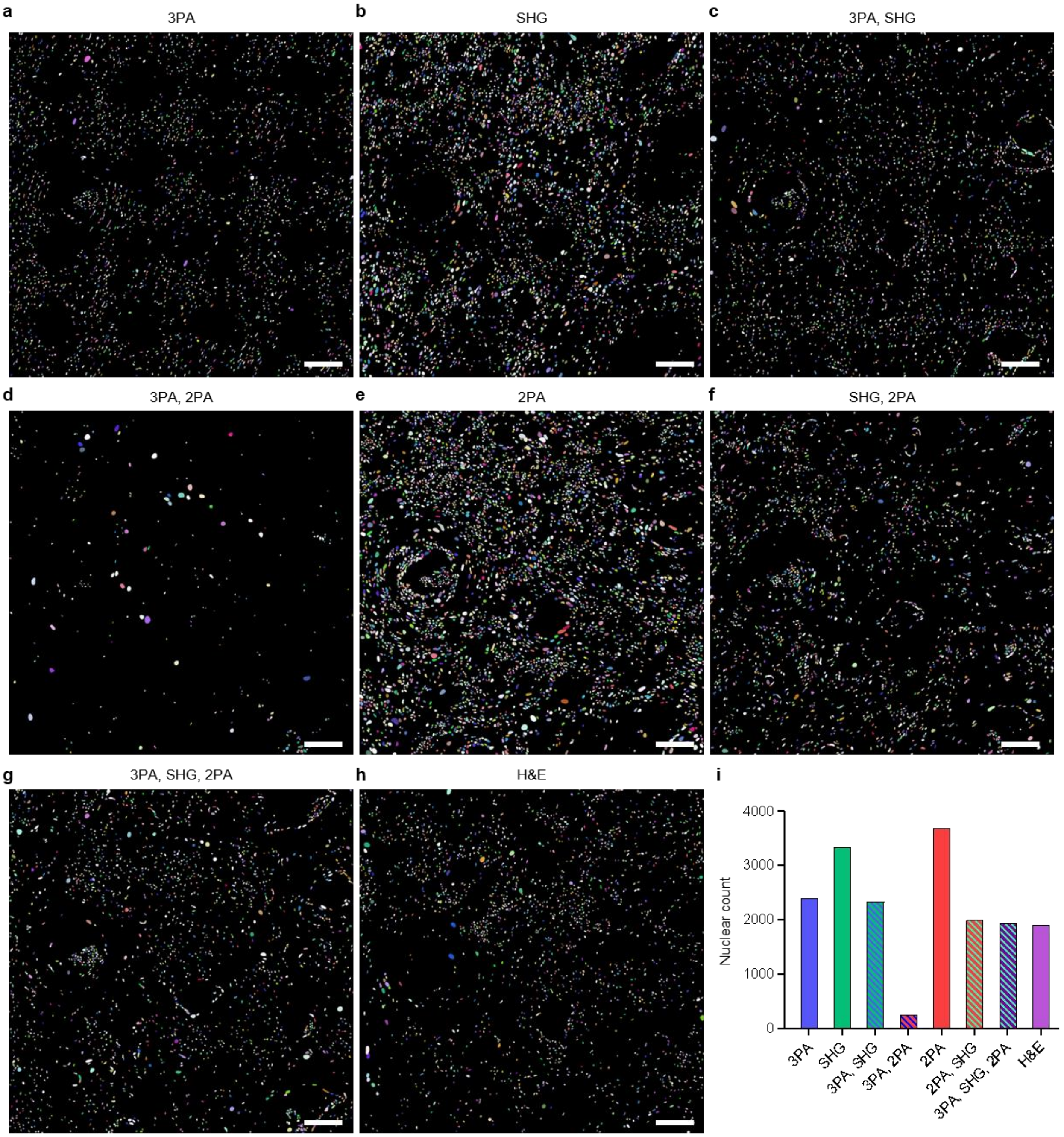

Fig. S8 Nuclear segmentation of different NOCH modalities.

a–h correspond to the seven modal permutations and combinations. **i**, Comparison of the nuclear count between different NOCH modalities and H&E stain for Stage IIIC. Scale bars, 200 μm.

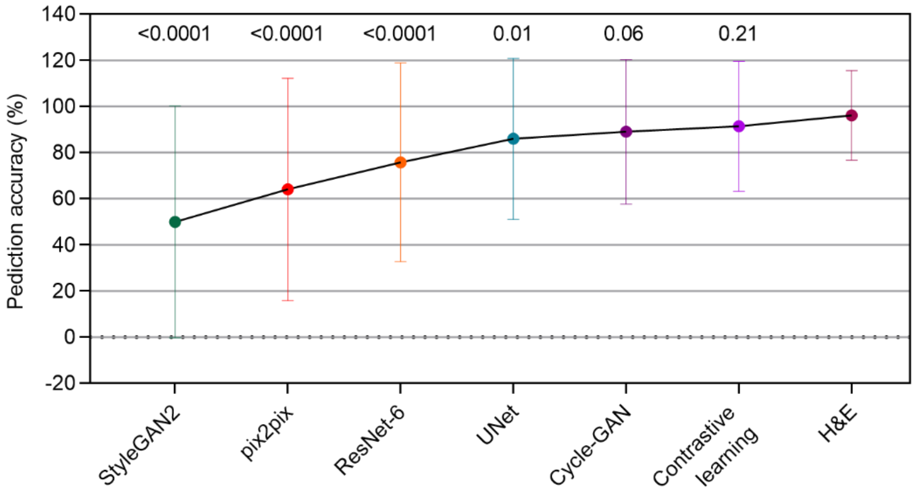

Fig. S9 Comparison of prediction accuracy of different models.

The *p* values of Wilcoxon matched-pairs signed rank test between the accuracy of the model results and that of the H&E reference were presented. n = 128. The results of our contrastive learning exhibit no significantly different with the reference.

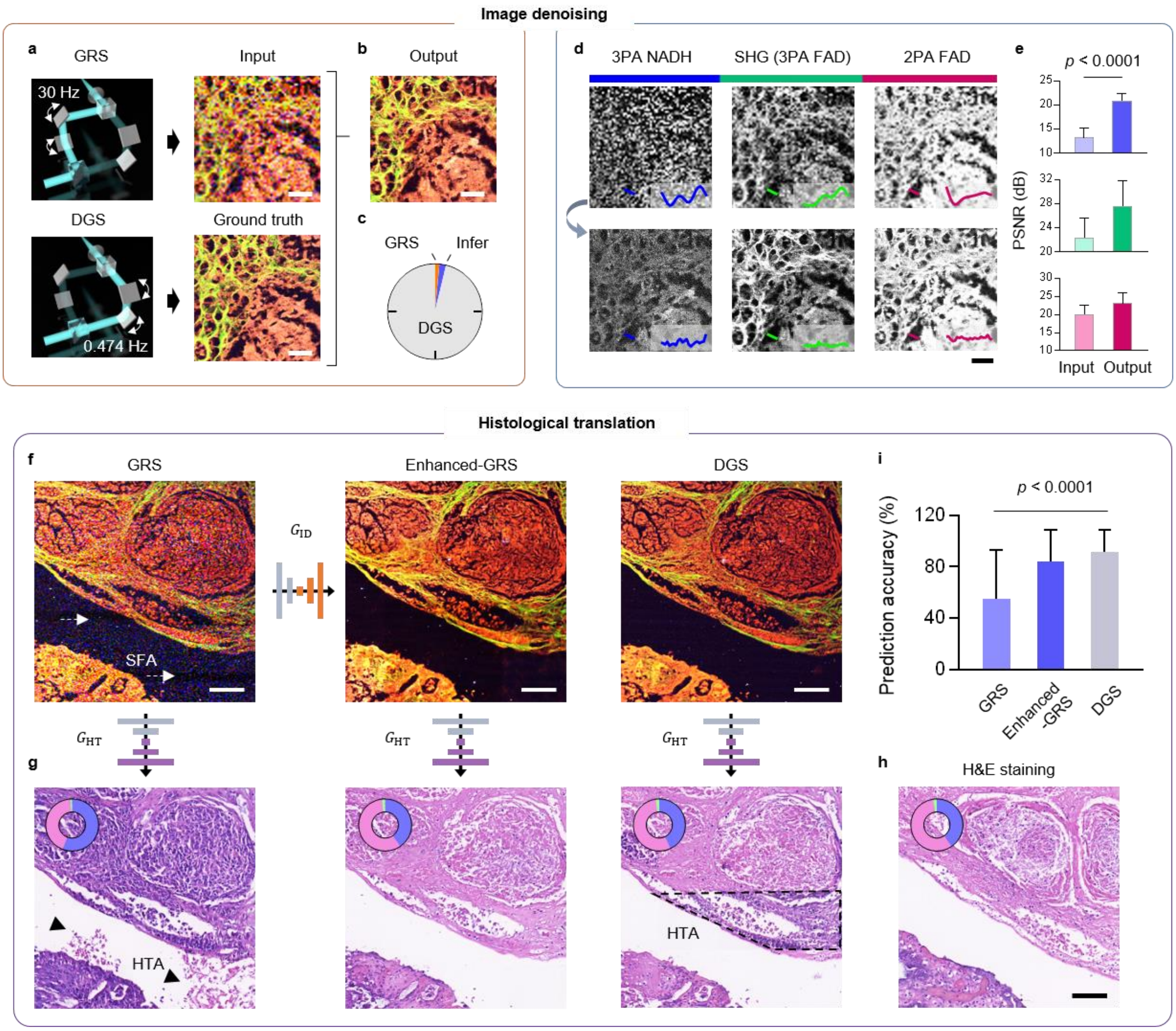

Fig. S10 High-speed acquisition and image denoising enables faster pathological diagnosis. **a**, GRS and DGS configurations and the acquisition images. **b**, Denoised image using contrastive learning. **c**, Comparison of GRS acquisition time, network inference time, and DGS acquisition time. **d**, Input (upper) and output (bottom) images of three nonlinear modalities. The color solid line in each image refers to the line of the shown cross-section of background and noise. **e**, PSNR of the input and output images with mean ± SD and two-tailed Wilcoxon matched-pairs test. n = 124. **f**, Left to right: GRS, network enhanced-GRS, and DGS images. **g**, The corresponding NOCH images. G_{ID} and G_{HT} represent the network generator of contrastive learning for image denoising and computational histology, respectively. **h**, H&E histologically stained image. Donuts in **g** and **h** indicate deconvolution portion of hematoxylin (blue), eosin (pink), and residual (cyan) components. **i**, Overall prediction accuracy for the pathological classes. A repeated measures one-way ANOVA, with the Geisser-Greenhouse correction was applied. Scale bars, 30 μm in **a**–**d** and 100 μm in **f**–**i**.

Table S1 Nuclear characteristics for Stage IIIC.

Modality	Nuclear number	Total area (μm^2)	Average size (μm)	% Area	Pixel mean
H&E	1897	70994.452	37.425	3.901	127.822
3PA	2396	83043.085	34.659	4.562	126.352
SHG	3332	180834.83	54.272	9.935	126.831
3PA-SHG	2332	89145.82	38.227	4.898	126.356
3PA-2PA	242	9880.233	40.827	0.543	126.778
2PA	3689	220291.95	59.716	12.103	125.282
2PA-SHG	1992	99984.747	50.193	5.493	126.954
3PA-SHG-2PA	1939	78792.006	40.635	4.329	127.821

Table S2 Size, speed and accuracy of different classification models.

**Model**	**FLOPs (G)** @batchsize: 1	**Parameters (M)**	**Speed (fps)** @544 × 544 px	**Total accuracy* (%)**
ResNet[6]	1.8	11.2	16	82.8
Res2Net[7]	4.3	23.6	13	82.0
Vision Transformer[8]	1.1	5.5	13	74.2
Swin Transformer[9]	8.5	48.8	11	60.9
CrossViT[10]	5.2	26.7	12	81.2
Inception-v4[11]	12.3	41.2	11	81.2
Xception[12]	9.3	24.9	12	87.5
ConvNeXt[13]	8.7	49.4	10	69.5
HRNet[14]	1.6	11.1	14	82.8
SKResNet[15]	1.8	11.4	15	85.9
SEResNet[16]	1.8	11.2	15	84.4
EfficientNet[17]	0.4	3.4	15	85.9
NEST[18]	9.4	37.4	12	86.7
DPN[19]	2.3	11.7	14	86.7
DLA[20]	3.0	15.2	15	89.8
DLA-Res2Net[7, 20]	4.2	19.8	13	85.9
**DLA-Res2Next[7, 20]**	**3.5**	**16.0**	**14**	**91.4**

*These networks were trained on the H&E histological images and tested on the NOCH data.

Supplementary references

1. Li, X.Y. et al. Unsupervised content-preserving transformation for optical microscopy. *Light: Science & Applications* **10**, 44 (2021).
2. Zhu, J., Park, T., Isola, P. & Efros, A.A. Unpaired Image-to-Image Translation using Cycle-Consistent Adversarial Networks *in IEEE International Conference on Computer Vision (ICCV)*. (eds. K. Ikeuchi, G. Medioni & M. Pelillo) 2242-2251 (Venice, Italy; 2017).
3. Ihle, S.J. et al. Unsupervised data to content transformation with histogram-matching cycle-consistent generative adversarial networks. *Nature Machine Intelligence* **1**, 461-470 (2019).
4. Shen, B.L. et al. Label-free whole-colony imaging and metabolic analysis of metastatic pancreatic cancer by an autoregulating flexible optical system. *Theranostics* **10**, 1849-1860 (2020).
5. Schmidt, U., Weigert, M., Broaddus, C. & Myers, G. Cell Detection with Star-Convex Polygons *in Medical Image Computing and Computer Assisted Intervention – MICCAI 2018*. (eds. A.F. Frangi, J.A. Schnabel, C. Davatzikos, C. Alberola-López & G. Fichtinger) 265-273 (Springer International Publishing, Cham; 2018).
6. He, K., Zhang, X., Ren, S. & Sun, J. Deep Residual Learning for Image Recognition *in 2016 IEEE Conference on Computer Vision and Pattern Recognition (CVPR)* 770-778 (2016).
7. Gao, S.H. et al. Res2Net: A New Multi-Scale Backbone Architecture. *IEEE Trans Pattern Anal Mach Intell* **43**, 652-662 (2021).
8. Dosovitskiy, A. et al. An image is worth 16x16 words: Transformers for image recognition at scale. *arXiv preprint* **2010.11929** (2020).
9. Liu, Z. et al. Swin Transformer: Hierarchical Vision Transformer using Shifted Windows *in 2021 IEEE/CVF International Conference on Computer Vision (ICCV)* 9992-10002 (2021).
10. Chen, C.F.R., Fan, Q. & Panda, R. CrossViT: Cross-Attention Multi-Scale Vision Transformer for Image Classification *in 2021 IEEE/CVF International Conference on Computer Vision (ICCV)* 347-356 (2021).
11. Szegedy, C., Ioffe, S., Vanhoucke, V. & Alemi, A.A. Inception-v4, Inception-ResNet and the Impact of Residual Connections on Learning *in Conference on Artificial Intelligence, AAAI*. (eds. S. Singh & S. Markovitch) 4278-4284 (AAAI Press, San Francisco; 2017).
12. Chollet, F. Xception: Deep Learning with Depthwise Separable Convolutions *in 2017 IEEE Conference on Computer Vision and Pattern Recognition (CVPR)* 1800-1807 (2017).

13. Liu, Z. et al. A ConvNet for the 2020s *in 2022 IEEE/CVF Conference on Computer Vision and Pattern Recognition (CVPR)* 11966-11976 (2022).
14. Wang, J. et al. Deep High-Resolution Representation Learning for Visual Recognition. *Ieee T Pattern Anal* **43**, 3349-3364 (2021).
15. Li, X., Wang, W., Hu, X. & Yang, J. Selective Kernel Networks *in 2019 IEEE/CVF Conference on Computer Vision and Pattern Recognition (CVPR)* 510-519 (2019).
16. Jie, H., Li, S., Gang, S. & Albanie, S. Squeeze-and-Excitation Networks. *Ieee T Pattern Anal* **42**, 2011–2023 (2017).
17. Tan, M. & Le, Q. Efficientnet: Rethinking model scaling for convolutional neural networks *in International conference on machine learning* 6105-6114 (PMLR, 2019).
18. Zhang, Z. et al. Nested hierarchical transformer: Towards accurate, data-efficient and interpretable visual understanding *in Proceedings of the AAAI Conference on Artificial Intelligence*, Vol. 36 3417-3425 (2022).
19. Chen, Y. et al. Dual path networks. *Advances in neural information processing systems* **30** (2017).
20. Yu, F., Wang, D., Shelhamer, E. & Darrell, T. Deep Layer Aggregation *in 2018 IEEE/CVF Conference on Computer Vision and Pattern Recognition* 2403-2412 (2018).
